# Comparative Genomics Reveal the Animal-Associated Features of the *Acanthopleuribacteraceae* Bacteria, and Description of *Sulfidibacter corallicola* gen. nov., sp., nov.

**DOI:** 10.3389/fmicb.2022.778535

**Published:** 2022-01-31

**Authors:** Guanghua Wang, Yuanjin Li, Jianfeng Liu, Biao Chen, Hongfei Su, Jiayuan Liang, Wen Huang, Kefu Yu

**Affiliations:** ^1^Guangxi Key Laboratory on the Study of Coral Reefs in the South China Sea, Nanning, China; ^2^Coral Reef Research Center of China, Guangxi University, Nanning, China; ^3^School of Marine Sciences, Guangxi University, Nanning, China

**Keywords:** animal acidobacteria, *Acanthopleuribacteraceae*, sulfhydrogenase, eicosapentaenoic acid (EPA), *Sulfidibacter corallicola*

## Abstract

Members of the phylum *Acidobacteria* are ubiquitous in various environments. Soil acidobacteria have been reported to present a variety of strategies for their success in terrestrial environments. However, owing to lack of pure culture, information on animal-associated acidobacteria are limited, except for those obtained from 16S rRNA genes. To date, only two acidobacteria have been isolated from animals, namely strain M133^T^ obtained from coral *Porites lutea* and *Acanthopleuribacter pedis* KCTC 12899^T^ isolated from chiton. Genomics and physiological characteristics of strain M133^T^ and *A. pedis* KCTC 12899^T^ were compared with 19 other isolates (one strain from each genus) in the phylum *Acidobacteria*. The results revealed that strain M133^T^ represents a new species in a new genus in the family *Acanthopleuribacteraceae*. To date, these two *Acanthopleuribacteraceae* isolates have the largest genomes (10.85–11.79 Mb) in the phylum *Acidobacteria*. Horizontal gene transfer and gene duplication influenced the structure and plasticity of these large genomes. Dissimilatory nitrate reduction and abundant secondary metabolite biosynthetic gene clusters (including eicosapentaenoic acid *de novo* biosynthesis) are two distinct features of the *Acanthopleuribacteraceae* bacteria in the phylum *Acidobacteria*. The absence of glycoside hydrolases involved in plant polysaccharide degradation and presence of animal disease-related peptidases indicate that these bacteria have evolved to adapt to the animal hosts. In addition to low- and high-affinity respiratory oxygen reductases, enzymes for nitrate to nitrogen, and sulfhydrogenase were also detected in strain M133^T^, suggesting the capacity and flexibility to grow in aerobic and anaerobic environments. This study highlighted the differences in genome structure, carbohydrate and protein utilization, respiration, and secondary metabolism between animal-associated acidobacteria and other acidobacteria, especially the soil acidobacteria, displaying flexibility and versatility of the animal-associated acidobacteria in environmental adaption.

## Introduction

Bacteria belonging to the phylum “Acidobacteria” are ubiquitous in various environments, including freshwater mud (Liesack et al., 1994; Zimmermann et al., 2012), hot spring microbial mats (Losey et al., 2013), metal-rich acidic waters (Falagán et al., 2017), various soils (Barns et al., 1999; Quaiser et al., 2003; Chanal et al., 2006; Vieira et al., 2017; Kalam et al., 2020), and animals (O’Connor-Sánchez et al., 2014; Liang et al., 2017; Laport et al., 2019). Soil acidobacteria, benefiting from abundant pure cultures (Kishimoto et al., 1991; Eichorst et al., 2007; Kulichevskaya et al., 2010, 2014; Pankratov and Dedysh, 2010; Okamura et al., 2011; Dedysh et al., 2012; Losey et al., 2013; Crowe et al., 2014; Tank and Bryant, 2015; Vieira et al., 2017) and genome sequencing, have been comprehensively studied in biogeographic patterns or survival strategies (Navarrete et al., 2015; Eichorst et al., 2018; Ivanova et al., 2020), metabolism of carbon (de Chaves et al., 2019) and sulfur (Hausmann et al., 2018), and benefits to plants (Kielak et al., 2016; Ivanova et al., 2020; Kalam et al., 2020). However, to date, *Acanthopleuribacter pedis* KCTC 12899^T^ is the only described acidobacterium isolated from animals (chiton) (Fukunaga et al., 2008). Recently, another acidobacterial isolate, strain M133^T^, was isolated from stony coral *Porites lutea*. Bacterial species belonging to other phyla have been demonstrated to be essential for animal development, metamorphosis, nutrition, and defense against pathogens or predator (McFall-Ngai et al., 2013; Perreau and Moran, 2022). However, since pure cultures are lacking, the roles of animal-associated acidobacteria are still unclear. Therefore, this study aimed to identify the animal-associated characteristics of acidobacteria using comparative genomic and high-throughput sequencing analyses based on these two strains.

## Materials and Methods

### Cultivation, Genome Sequencing, and Assembly

*Acanthopleuribacter pedis* KCTC 12899^T^ isolated from a chiton (*Acanthopleura japonica*) in Japan was obtained from the Korean Collection for Type Cultures (KCTC). This strain grows well on both Marine Agar 2216 (BD) and MA/10 [1/10 marine broth powder (BD), 3/4 natural sea water, agar powder 1.2 g/L].

Strain M133^T^ was isolated using serial 1/10 dilution plating on MA/10 from coral *Porites lutea* collected from the Weizhou Island (109°08′35″ E, 21°03’42″ N), China. This strain grows well on MA/10, but not full-strength Marine Agar 2216(BD).

The whole bacterial biomass was collected from MA/10 or MB/10 [1/10 strength marine broth (BD), 3/4 natural sea water] if not indicated.

For the genome analysis, strain M133^T^ and *A. pedis* KCTC 12899^T^ were incubated in MB/10 (1/10 strength marine broth [BD], 3/4 natural sea water) at 25°C with shaking. After 5 days of incubation, the biomass was collected and used for DNA extraction. Genomic DNA was extracted using the SDS method (Lim et al., 2016). The extracted DNA was visualized using agarose gel electrophoresis and quantified using the Qubit^®^ 2.0 Fluorometer (Thermo Scientific). The whole genome of strain M133 was sequenced using the PacBio Sequel platform and Illumina NovaSeq PE150, while the genome of *A. pedis* KCTC 12899^T^ was sequenced using the Illumina NovaSeq PE150. Genomes sequencing was performed by the Beijing Novogene Bioinformatics Technology Co., Ltd. Libraries for single-molecule real-time (SMRT) sequencing were constructed with an insert size of 10 kb using the SMRT bell TM Template kit (v1.0). Meanwhile, libraries for Illumina sequencing were generated using the NEBNext^®^ Ultra™ DNA Library Prep Kit (NEB, United States) following the manufacturer’s recommendations. For Illumina NovaSeq PE150 sequencing, raw data were filtered using readfq (v10) to obtain clean data. The specific processing steps were as follows: (1) removal of reads containing low-quality bases (mass value ≤ 20) over 40%; (2) removal of reads containing ≥ 10% N; and (3) removal of reads overlapping with adapters ≥ 15 bp. Clean data were assembled using the SOAP *de novo* (Luo et al., 2012), SPAdes (Bankevich et al., 2012), and Abyss (Simpson et al., 2009), and the results of these three software were integrated with CISA (Lin and Liao, 2013). For the PacBio Sequel platform, low-quality reads (less than 500 bp) were filtered, and clean reads were assembled using SMRT Link (Ardui et al., 2018) and corrected with Illumina data. Finally, the sequencing depth of *A. pedis* KCTC 12899^T^ was 55 × with a Q20 of 97%. For strain M133, the PacBio sequencing depth was 236 × , the Illumina sequencing depth was 73 × with a Q20 of 98%.

### Genome Annotation and Comparative Analysis

Gene search and annotation were performed using GeneMarkS (Besemer et al., 2001) and RASTtk (Aziz et al., 2008) in Kbase (Arkin et al., 2018). Metabolism pathways were annotated using the KASS server (Kanehisa et al., 2004). Annotation of COGs was performed using WebMGA (Wu et al., 2011). Peptidases were annotated using the Hotpep-protease (Bush, 2020) based on the Merops database. Carbohydrate-active enzyme annotation was conducted using the dbCAN meta server (Zhang et al., 2018). GIs and prophages were detected using the IslandViewer 4 webserver (Bertelli et al., 2017). Phaster (Arndt et al., 2016), respectively. The insert sequences were detected using IsSaga (Varani et al., 2011). The results of the detected prophages and insert sequences were manually checked with the GenBank annotations. Meanwhile, the pan-genome was calculated using OrthoMCL (2.0) with default parameters in the KBase server (Arkin et al., 2018). Duplicated genes for each genome were calculated from the pan-genome orthologous results. Intra-genome collinearity analysis was performed using MCScanX (Wang et al., 2012). Secondary metabolite biosynthetic gene clusters (BGCs) were analyzed using the online secondary metabolite search tool antiSMASH (Blin et al., 2021). Gene or gene cluster was visualized using Circos (Krzywinski et al., 2009). The average nucleotide identity was calculated using the online ANI calculator (Yoon et al., 2017). Lastly, the average amino acid identity was calculated using the EzAAI (Kim et al., 2021).

### Transcriptome Analysis

H_2_S and N_2_ production pathways were checked using transcriptome analysis. For transcriptome analysis, strain M133^T^ was firstly incubated in shaking MB/10 at 25°C. After 3 days of incubation, 50 ml of inoculum was inoculated into two bottles of 200 ml MB, respectively. Collected one bottle of M133^T^ using centrifugation after 1 day of shaking incubation at 25°C. Then another bottle of M133^T^ was changed to static incubation after 1 day of shaking incubation at 25°C until the emergence of black particles. Collected the black-stained biomass using centrifugation. Biomass was frozen using liquid nitrogen and stored at –70°C until RNA extraction. RNA was extracted using the RNAprep Pure Cell/Bacteria Kit (Tiangen, Beijing). Sequencing libraries were generated using NEBNext^®^ UltraTM RNA Library Prep Kit for Illumina^®^ (NEB, United States) following the manufacturer’s recommendations. Sequencing was performed using the Illumina Novaseq platform at the Beijing Novogene Bioinformatics Technology Co., Ltd. Clean reads were obtained after removing adapter and low quality reads. Reads containing N were removed; Reads containing low-quality bases (mass value ≤ 20) over 50% were removed. Sequencing depth was 95×, and quality score Q20 was 98%. Bowtie2-2.2.3 was used in mapping reads to reference genome (Langmead and Salzberg, 2012). Rockhopper was used to identify novel genes, operon, TSS, TTS and Cis-natural antisense transcripts (McClure et al., 2013). HTSeq was used to count the reads numbers mapped to each gene (Anders et al., 2015), and then FPKM of each gene was calculated based on the length of the gene and reads count mapped to this gene. When FPKM < 1, no expression is considered. Prior to differential gene expression analysis, for each sequenced library, the read counts were adjusted by edgeR program package (Robinson et al., 2010). Differential expression analysis of two conditions was performed using the DEGSeq R package (Wang et al., 2010).

### Polyphasic Identification of Strain M133^T^

Taxonomic assignment of strain M133^T^ was following the standard procedure of prokaryotic microbe identification, namely phenotypic analysis, phylogenetic analysis (Felsenstein, 1981; Saitou and Nei, 1987; Swofford, 1993; Kumar et al., 2016), phylogenomic analysis (Na et al., 2018) and chemotaxonomic analysis as done in Wang et al. (2020).

### Coral Autotrophic Incubation

Coral *P. lutea* was collected from Weizhou Island and incubated in an aquarium at Guangxi University. For the autotrophic experiments, coral fragments were incubated in axenic natural seawater under natural light at room temperature (approximately 25°C) without feeding. During autotrophic incubation, coral fragments were washed with axenic natural seawater once a week to replace the seawater used during incubation. For the preparation of axenic natural seawater, natural seawater was sterilized at 121°C for 20 min, followed by the injection of axenic air for 20 min to restore the carbonate system.

### Bacterial Composition Analysis

Coral holobiont DNA was extracted from 1 cm × 1 cm coral tissue using the TaKaRa MiniBEST Universal Genome DNA extract kit (v5.0). The bacterial community was analyzed using 16S rRNA gene libraries generated with the 338F (5′-ACTCCTACGGGAGGCAGCAG-3′) and 806R (5′-GGACTACHVGGGTWTCTAAT-3′) primers following the procedure described by Chen et al. (2021).

### Genomes of Acidobacteria From the GenBank

*Acidipila rosea* DSM 103428^T^ (GCF_004339725), “*Acidisarcina polymorpha*” SBC82 (GCF_003330725), *Acidobacterium capsulatum* ATCC 51196^T^ (GCF_000022565), *Bryocella elongata* DSM 22489^T^ (GCF_900108185), *Candidatus* Solibacter usitatus Ellin6076 (GCF_000014905), *Edaphobacter modestus* DSM 18101^T^ (GCF_004217555), *Granulicella pectinivorans* DSM 21001^T^ (GCF_900114625), *Occallatibacter savannae* AB23^T^ (GCF_003131205), *Silvibacterium bohemicum* S15^T^ (GCF_001006305), *Terracidiphilus gabretensis* S55^T^ (GCF_0014 49115), *Terriglobus roseus* DSM 18391^T^ (GCF_000265425), *Terriglobus saanensis* SP1PR4^T^ (GCF_000179915), *Bryobacter aggregatus* MPL3^T^ (GCF_000702445), *Paludibaculum fermentans* P105^T^ (GCF_015277775), *Pyrinomonas methylaliphatogenes* K22^T^ (GCF_000820845), *Chloracidobacterium thermophilum* B^T^ (GCF_000226295), *Holophaga foetida* DSM 6591^T^ (GCF_000242615), *Geothrix fermentans* DSM 14018^T^ (GCF_000428885), *Thermoanaerobaculum aquaticum* MP-01^T^ (GCF_000687145), and *Luteitalea pratensis* DSM 100886^T^ (GCF_001618865).

### Sequence Accession Numbers

The GenBank/EMBL/DDBJ accession numbers for the 16S rRNA gene sequence and the whole genome sequence of strain M133^T^ are MN908335 and CP071793, respectively. The GenBank/EMBL/DDBJ accession number for the whole genome sequence of strain *Acanthopleuribacter pedis* KCTC 12899^T^ is JAFREP000000000. The raw 16S rRNA gene reads of bacteria from the coral *P. lutea* are deposited in the NCBI Sequence Read Archive database (SRA) under BioProjects PRJNA786650 and PRJNA787388.

## Results and Discussion

### General Genome Features

The complete genome sequence of strain M133^T^ is 11,786,365 bp long with a G + C content of 60.2 mol%. The genome contains three 16S-23S-5S rRNA operons and 57 tRNA genes. A total of 6,923 protein-coding genes were identified. Meanwhile, the whole genome sequence of *A. pedis* KCTC 12899^T^ consists of 10,848,621 bp genome sequences obtained from 98 scaffolds, with a G + C content of 57.3 mol%. Five 5S rRNA, one 16S rRNA, one 23S rRNA, and 65 tRNA genes were identified. In total, 6,716 protein-coding genes were identified. Other acidobacterial genome sequences of type or *Candidatus* species with genome sizes of 2.66 to 9.97 Mb were obtained from GenBank (Table 1).

**TABLE 1:** Features of selected acidobacterial genomes.

Strains	Genome size	Biosynthetic gene clusters		Coding gene duplication			Genomic islands		Prophage No.	Insert sequence No.	BGCs + CGDs + GIs
		No.	Size	No.	Size	Increment	No.	Size			
M133^T^	11.79	43	2.67	1320	3.11	2.34	44	0.87	4	87	5.12
*A. pedis* KCTC 12899^T^	10.85	40	2.18	1443	3.12	2.27	17	0.48	1	13	5.78
*G. fermentans* DSM 14018^T^	3.29	1	0.01	352	0.34	0.23	14	0.36	0	51	0.71
*H. foetida* DSM 6591^T^	4.13	0	0	816	0.91	0.57	17	0.28	2	61	1.19
*T. aquaticum* MP-01^T^	2.66	1	0.05	218	0.28	0.16	5	0.08	0	2	0.41
*L. pratensis* DSM 100886^T^	7.48	4	0.16	1365	1.69	1.02	34	0.39	2	51	2.10
*C. thermophilum* B^T^	3.65	4	0.15	454	0.63	0.38	9	0.12	3	41	0.73
*P. methylaliphatogenes* K22^T^	3.79	2	0.07	394	0.52	0.29	13	0.20	4	21	0.79
*A. rosea* DSM 103428^T^	4.21	6	0.25	366	0.54	0.30	22	0.29	1	12	1.08
*T. roseus* DSM 18391^T^	5.23	7	0.17	1126	1.41	0.80	11	0.36	5	7	1.83
*T. gabretensis* S55^T^	5.35	9	0.3	550	0.79	0.46	65	0.66	2	15	1.75
*S. bohemicum* S15^T^	6.46	13	0.41	1013	1.06	0.60	20	0.33	2	19	1.80
*O. savannae* AB23^T^	6.28	4	0.1	916	1.10	0.66	52	0.64	1	8	1.84
*G. pectinivorans* DSM 21001^T^	5.28	7	0.16	628	0.89	0.56	33	0.32	3	7	1.37
*E. modestus* DSM 18101^T^	7.45	10	0.31	1446	1.80	1.12	43	0.57	2	231	2.00
*B. elongate* DSM 22489^T^	5.67	8	0.27	665	0.62	0.59	18	0.25	1	10	1.14
*A. capsulatum* ATCC 51196^T^	4.13	6	0.22	317	0.49	0.28	8	0.46	1	31	1.01
“A. polymorpha” SBC82	7.6	12	0.42	1520	1.83	1.12	14	0.27	4	92	2.32
*P. fermentans* P105^T^	9.48	8	0.24	1793	2.57	1.61	53	0.57	3	36	3.21
*B. aggregatus* MPL3^T^	5.75	2	0.05	831	1.13	0.73	17	0.39	1	70	1.17
*Can*. S. usitatus Ellin6076	9.97	7	0.28	2076	3.01	1.92	48	0.72	2	91	3.70

*NO., number; GIs, Genomic islands; BGCs, secondary metabolite biosynthetic gene clusters; CGDs, Coding gene duplications. Size unit (Mb). Numbers with underline just as reference because of incomplete genome sequencing.*

### Genome Structure and Plasticity

Based on the available sequences in GenBank, acidobacterial genome sizes range from 0.55 to 11.88 Mb, of which the completed genome sizes range from 2.32 to 11.79 Mb. The largest acidobacterial genome before this study belonged to *Candidatus* S. usitatus Ellin6076 (9.97 Mb) that was proposed to arise via horizontal gene transfer, followed by widespread small-scale gene duplication (Challacombe et al., 2011; Challacombe and Kuske, 2012). Thus far, strain M133 has the largest genome (11.79 Mb) among the completely sequenced acidobacteria, followed by *A. pedis* KCTC 12899^T^ (10.85 Mb).

To explore the mechanism underlying large genomes, the pan-genome was calculated using 21 selected acidobacteria. Proteins from the 21 selected acidobacteria were clustered into 29,763 families, of which only 418 families belonged to the core genome, suggesting the high diversification of these acidobacteria. The genome of strain M133^T^ contains 1,355 unique protein families (species-specific; total length of encoding genes is approximately 1.69 Mb) and 4,737 shared protein families (orthologs). The core genome of strain M133^T^ and *A. pedis* KCTC 12899^T^ is approximately 7.44 Mb, encoding 4,246 families of proteins, which is larger than most of the selected acidobacterial genomes (Table 1), suggesting that the last common ancestor of strain M133^T^ and *A. pedis* KCTC 12899^T^ had already obtained a large genome.

**FIGURE 1:**
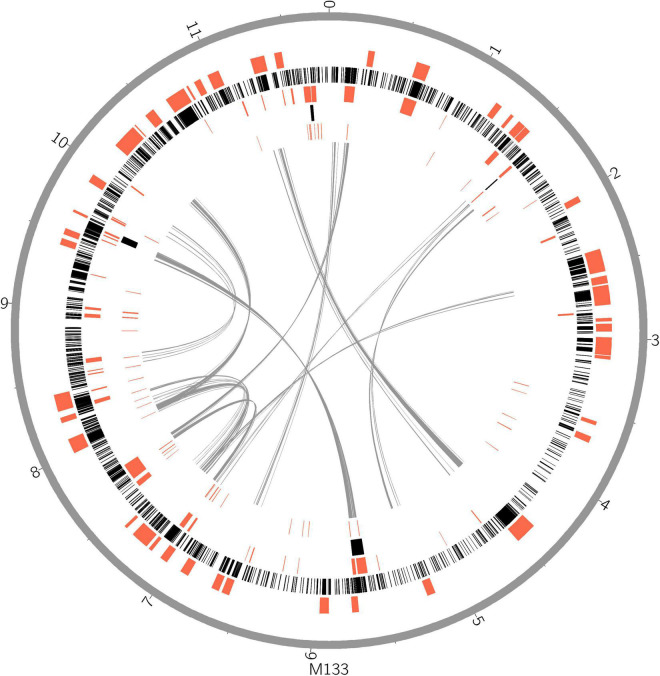
Horizontal gene transfer and gene duplication shaped the large genome of strain M133^T^. From outer circle to inner, genome scale (gray), biosynthetic gene clusters (red), coding gene duplications (black), genomic islands, insert sequences, intra genome collinearity were displayed. Genome size unit, Mb.

Horizontal gene transfer (HGT) events are important in acidobacteria for carbohydrate utilization (Naumoff and Dedysh, 2012; Naumoff, 2016), heavy metal resistance, iron uptake, secondary metabolism, and antibiotic resistance (Gonçalves and Santana, 2021). To explore HGT events, genomic islands (GIs) were explored in selected complete acidobacterial genomes (data from draft genomes were listed as references, which may underestimate the actual GIs, prophage, and insert sequence level). The quantities of GIs were highly variable among the genomes, while no habitant or taxonomic dependence was found (Table 1). The total lengths of GIs in an individual genome range from 0.08 to 0.87 Mb, accounting for 3.01–12.34% of the genome. Mobile genetic elements (MGEs), agents that affect DNA movement in HGT (Frost et al., 2005), were also analyzed. A total of 44 prophages were detected in the selected genomes, wherein 1–4 prophages were detected in an individual genome (Table 1). Interestingly, insert sequences, which were detected in all 21 genomes, were more abundant than prophages in acidobacterial genomes, ranging from 2–231 in an individual genome (Table 1). After manual verification, 44 GIs, 4 prophages, and 87 insert sequences were detected in the genome of strain M133^T^. The total length of 44 GIs accounted for approximately 7.38% of the genome. Structurally, all four prophages had corresponding GIs in the genome. A few insert sequences had no corresponding GIs (Figure 1), indicating that these insert sequences may be species-specific. Some prophages and insert sequences co-localized in the same genomic region (Figure 1), suggesting gene transfer and/or recombination events between mobile genetic elements (Eichorst et al., 2018).

Gene duplications are common and highly variable among the selected acidobacterial genomes. Duplicated genes range from 218 to 2,076 for an individual genome, with a total length of 0.28–3.12 Mb, which accounts for 10.33–30.19% of the genome (Table 1). The total length of duplicated genes in the genome of strain M133^T^ is 3.11 Mb, which accounts for 26.38% of the genome. The duplicated genes in strain M133^T^ were detected genome-wide (Figure 1), mainly functioning in membrane transport, secondary metabolite biosynthesis, MGEs, and others (Supplementary Table 1). Furthermore, these duplicated genes suggest that there were also widespread gene duplications after HGT events in strain M133^T^ similar to *Can.* S. usitatus Ellin6076 (Challacombe et al., 2011). In addition to MGEs, some species-specific genes were duplicated, such as the rRNA genes (Supplementary Figure 1). In total, three rRNA operons were detected in the genome of strain M133, with one orthologous operon with tRNA-Ile-tRNA-Ala inserted between the 16S and 23S rRNA genes, which is consistent with most of the acidobacteria except for *Bryobacteraceae* (tRNA-Ala-tRNA-Ile). The other two operons have lost the tRNA-Ile-tRNA-Ala insertion and are located in different strands. The 5S rRNA end of the reverse-strand operon is linked to a site-specific tyrosine recombinase, indicating that the duplicated rRNA operons were from site-specific recombination.

Biosynthetic gene clusters (BGCs) usually result from the combination of HGT, duplication, and rearrangement events during complex evolutionary processes (Lopez, 2003; Gallagher and Jensen, 2015; Boutanaev and Osbourn, 2018). The results indicate that BGCs are as large as GIs in strain M133^T^ (Figure 1) and *A. pedis* KCTC 12899^T^, accounting for 22.6% and 20% of the genome, respectively (Table 1). For other acidobacteria, BGCs account for less than 6.5%. Some prophages and/or insert sequences were co-localized with BGCs in the same genome regions (Figure 1), suggesting that MGEs have affected the evolution of these BGCs.

The total length of GIs, duplicated genes, and BGCs (plus overlaps) is 5.12 Mb in the genome of strain M133^T^, which accounts for 43.43% of the genome size. Meanwhile, the total length of these in *Can.* S. usitatus Ellin6076 and *P. fermentans* P105^T^ are 3.70 and 3.21 Mb, accounting for 37.11% and 33.86% of the genome, respectively (Table 1). Therefore, HGT and gene duplication have impacted the long-term evolution of these large genome acidobacteria.

### General Metabolism Analysis

The quantity of clusters of orthologous groups (COGs) for acidobacteria ranged from 1260 to 1962, and larger genomes encoded more COGs (Supplementary Table 2). According to N class COGs of *A. capsulatum* ATCC 51196^T^ (Kishimoto et al., 1991), 17 of the 21 selected acidobacteria should be motile (Supplementary Table 2), although some of them were reported to be non-motile (Liesack et al., 1994; Eichorst et al., 2007; Kulichevskaya et al., 2010, 2014; Pankratov and Dedysh, 2010; Okamura et al., 2011; Dedysh et al., 2012; Crowe et al., 2014; Vieira et al., 2017). *C. thermophilum* B^T^, *G. fermentans* DSM 14018^T^, and *T. aquaticum* MP-01^T^ contain 2–7 families of N-class COGs, which are consistent with the non-motile reports (Coates et al., 1999; Losey et al., 2013; Tank and Bryant, 2015).

Meanwhile, the quantities of G-class COGs varied among habitats (Supplementary Table 2), which decreased in the sequence of soil acidobacteria, animal-associated acidobacteria (*A. pedis* KCTC 12899^T^ and strain M133^T^), and microaerophilic or anaerobic acidobacteria (*H. foetida* DSM 6591^T^, *C. thermophilum* B^T^, *G. fermentans* DSM 14018^T^, and *T. aquaticum* MP-01^T^), indicating the adaptive carbohydrate utilization abilities of these strains (Ward et al., 2009; de Castro et al., 2013; Belova et al., 2018; Eichorst et al., 2018; Kalam et al., 2020). The Q-class of COGs (Supplementary Table 2) indicated that strain M133^T^ and *A. pedis* KCTC 12899^T^ harbors genes involved in secondary metabolite biosynthesis, transport, and catabolism compared with other acidobacteria (details are displayed in the section “Secondary Metabolism”).

### Carbohydrates Metabolism

Carbohydrate-active enzymes (CAZymes) prediction results (Supplementary Table 3) revealed that facultative anaerobic *P. fermentans* P105^T^ and *Can.* S. usitatus Ellin6076 have the most abundant families of CAZymes (111–112), followed by aerobic soil acidobacteria (75–103), animal-associated acidobacteria (56–63), and anaerobic bacteria (23–35). Glycoside hydrolases, which are involved in hydrolysis and/or rearrangement of glycosidic bonds, contributed to the differences in CAZyme abundance among the genomes (Supplementary Table 3). Soil acidobacteria have 40–71 families of glycoside hydrolases, while animal-associated and anaerobic acidobacteria have very few glycoside hydrolase families (23–24 and 7–11, respectively). These results further confirmed the outstanding carbohydrate utilization ability of soil acidobacteria (Ward et al., 2009; de Castro et al., 2013; Belova et al., 2018; Eichorst et al., 2018; Kalam et al., 2020). Interestingly, only 48 CAZymes were detected in the aerobe *Bryobacter aggregatus* MPL3^T^ isolated from acidic *Sphagnum* peat bogs, of which 18 families belonged to glycoside hydrolases.

In this study, Acidobacteria are observed to be versatile in carbohydrate degradation. Genes encoding for chitinases (GH18, 23) involved in chitin degradation; α-amylases (GH57), glucoamylases (GH15), and glycogen/starch phosphorylases (GT35) in starch degradation; endoglucanases (GH51, 9, and 10) and β-glucosidases (GH3) in cellulose degradation, and xylanases (GH10, 51) in xylan degradation were detected in most of the acidobacterial genomes (Supplementary Table 3). Previously, some acidobacteria have been shown to degrade a range of carbohydrates. For example, soil bacteria *Silvibacterium bohemicum* S15^T^ (Lladó et al., 2016) and “Acidisarcina polymorpha” SBC82 (Belova et al., 2018) have been reported to degrade starch, cellulose, chitin, xylan, and pectin. Genes encoding carbohydrate hydrolases indicated that strain M133^T^ and *A. pedis* KCTC 12899^T^ can degrade chitin (mainly from the cell wall of fungi and the exoskeleton of arthropods) using N-acetylglucosaminidase (GH20, Meekrathok et al., 2021), but not pectin (mainly from plants) using polygalacturonases (GH28) and α-rhamnosidases (CBM67, GH106).

Furthermore, acidobacteria are versatile in polysaccharide biosynthesis. Starch is synthesized by the starch synthase and 1,4-alpha-glucan branching enzyme. Genes encoding starch synthases (GT5) were detected in 20 of the selected acidobacterial genomes, excluding *Geothrix fermentans* DSM 14018^T^, while those encoding the 1,4-alpha-glucan branching enzyme (CBM48 + GH13_9) were detected in only seven genomes (Supplementary Table 3). These observations indicate that most of the acidobacteria can synthesize amylose, while only a few (including strains M133 and *A. pedis* KCTC 12899^T^) can produce glycogen. In particular, *Acidobacteriaceae* cannot produce glycogen.

Cellulose is important in biofilm formation and cellular water holding and is synthesized by various cellulose synthases. To date, only the genomes of *A*. *capsulatum* and *Terriglobus saanensis* have been reported to biosynthesize cellulose using *bcs* operons (Kalam et al., 2020). Genomic detection using the Kbase platform and conserved domains search in GenBank indicated that seven acidobacteria in the family *Acidobacteriaceae*, namely *A*. *capsulatum* ATCC 51196^T^, *T. saanensis* SP1PR4^T^, *A. rosea* DSM 103428^T^, *B. elongate* DSM 22489^T^, *G. pectinivorans* DSM 21001^T^, *T. gabretensis* S55^T^, and “A. polymorpha” SBC82, contain *bcs* operons. The distribution of the *bcs* operon was variable in the genus *Edaphobacter*, and no *bcs* operon was detected in the genome of *E. modestus* DSM 18101^T^. Cellulose synthase was not detected in strain M133^T^ and *A. pedis* KCTC 12899^T^.

### Peptide Degradation

According to the catalog of MEROPS peptidase, the genomes of selected acidobacteria contain 45–95 families of peptidases (Supplementary Table 4). Species from the same family have similar numbers of peptidase families. The anaerobic *Holophagaceae foetida* DSM 6591^T^ has the lowest number of peptidase family (45). *Acidbacteriaceae* had 57–77 peptidase families, followed by animal-associated *Acanthopleuribacteraceae* (79–84), and *Bryobacteriaceae* has the highest number of peptidase families (79–95). Other acidobacteria have 62–87 families of peptidases.

Bacterial collagenase H (M09B), imelysin (M75), and MtfA peptidase (M90) were only detected in strain M133 and *A. pedis* KCTC 12899^T^ (Supplementary Table 4). Moreover, collagenase (U32), subtilisin (S08A), pappalysin-1(M438), astacin (M12A), adamalysin (M12B), serralysin (M10B), fungalysin (M36), lysostaphin (M23B), immune inhibitor A peptidase (M06), thermolysin (M04), and bacteriocin-processing peptidase (C39) were detected in strain M133^T^ and *A. pedis* KCTC 12899^T^. In particular, a high abundance of subtilisin (11–12) and thermolysin (8–11) coding sequences were detected in strain M133 and *A. pedis* KCTC 12899^T^ (Supplementary Table 4). Membrane dipeptidase (M19), carnosine dipeptidase II (M20F), glutamate carboxypeptidase (M28B), aminopeptidase M29 (M29), streptogrisin A (S01E), carboxypeptidase Y (S10), SpoIVB peptidase (S55), Xaa-Pro dipeptidyl peptidase (S15), and sedolisin (S53) were absent in strain M133^T^ and *A. pedis* KCTC 12899^T^, whereas they are widely detected in other acidobacteria.

### Secondary Metabolism

Genes encoding enzymes that involved in peptide assembly, regulation, resistance, and synthesis of secondary metabolites are usually physically clustered into biosynthetic gene clusters (BGCs, Medema et al., 2015). More than 40 BGCs were detected in genomes of strain M133^T^ and *A. pedis* KCTC 12899^T^, respectively (Table 1). According to the antiSMASH prediction, these BGCs may function in non-ribosomal peptides, polyketides, terpenes, thioamitides, arylpolyenes, and ranthipeptides biosynthesis. While other acidobacterial genomes contain only a few BGCs (0–13) (Table 1). Genes of non-ribosomal peptide synthetases and type I polyketide synthetases accounted for more than 40% of the BGCs in strain M133^T^ and *A. pedis* KCTC 12899^T^. The exact secondary metabolites of strain M133^T^ and *A. pedis* KCTC 12899^T^ need further study.

Bacterial long-chain polyunsaturated fatty acids (PUFAs) are synthesized by multi-domain protein complexes akin to type I iterative fatty acids and polyketide synthases (Shulse and Allen, 2011). The typical PUFA-synthesizing gene cluster was discovered in marine gammaproteobacteria, in which five genes, *pfaABCDE*, participated in the *de novo* biosynthesis of PUFAs (Shulse and Allen, 2011). EPA was detected in both strain M133^T^ and *A. pedis* KCTC 12899^T^ using MIDI gas chromatography, with EPA contents of 6.2% and 12.2%, respectively (Supplementary Table 5). Among the five genes in the EPA-synthesizing gene cluster, four genes (*pfaB* and *pfaC* were annotated as one gene: *pfaB/C*) were detected in strain M133^T^ and *A. pedis* KCTC 12899^T^, wherein three genes formed the cluster *pfaDAB/C*, while *pfaE* encoding for phosphopantetheinyl transferase was located far from this cluster (Supplementary Figure 2). This EPA biosynthesis cluster is much similar to those in deltaproteobacteria “*Desulfococcus oleovorans*” Hxd3 and *Sorangium cellulosum* So ce 56 (*S. bulgaricum* Soce 321^T^ was reported to produce EPA, Mohr et al., 2018), but not the EPA-producing gammaproteobacteria *Shewanella pealeana* ATCC 700345^T^ (Shulse and Allen, 2011). None of the *pfa* genes were detected in the other acidobacterial genomes.

### Respiration of *Acidobacteria*

There are several types of respiration electron acceptors in acidobacteria, such as oxygen, nitrate, Fe (III), Mn (IV), and sulfur. Most acidobacteria, mainly *Acidobacteriaceae*, are aerobic or microaerobic. The A-type cytochrome c oxidase was ubiquitous in *Acidobacteriaceae*, while the high-oxygen affinity cbb3-type cytochrome c oxidase and cytochrome bd ubiquinol oxidase were detected in only half of the selected genomes (Supplementary Table 6). This illustrates that some of *Acidobacteriaceae* can respond to microoxic concentrations (Morris and Schmidt, 2013; Eichorst et al., 2018). Meanwhile, only the cbb3-type terminal oxidase has been detected in the genome of the microaerophile *C. thermophilum* (Tank and Bryant, 2015). Both strain M133 and *A. pedis* KCTC 12899^T^ contain the A-type cytochrome c oxidase, cbb3-type cytochrome c oxidase, and cytochrome bd ubiquinol oxidase, suggesting that these two strains can survive under variable oxygen conditions.

Nitrate is an energy-efficient oxygen substitute under anaerobic conditions. However, it seems that acidobacteria are not adept at nitrate reduction. Genes for assimilatory nitrate reduction were detected in five of the 21 genomes, and those for nitrite reduction were detected in six genomes. Dissimilatory nitrate reduction genes were detected in four genomes, namely *G. ferrnentans* DSM 14018^T^, *T. aquaticum* MP-01^T^, *A. pedis* KCTC 12899^T^, and strain M133, while those for nitrite reduction were found in five genomes. These results may be due to a few assimilatory nitrate reductases involved in dissimilatory nitrogen metabolism, as suggested by Morozkina and Zvyagilskaya (2007); Eichorst et al. (2018). Denitrification genes *nirS* and *norBC* were detected in *G. ferrnentans* DSM 14018^T^, *T. aquaticum* MP-01^T^, *P. fermentans* P105^T^, *Luteitalea pratensis* DSM 100886^T^, and *A. pedis* KCTC 12899^T^, whereas the complete denitrification pathways, which included *napA*, *nirS*, *norBC*, and *nosZ* were detected only in strain M133^T^. Transcriptome analysis confirmed the expression of genes *nirS* (FPKM 2311: 1.2), *norB* (1364: 0.6), *norC* (856: 0), and *nosZ* (723: 1.3) under anaerobic conditions in strain M133^T^. API 20NE results also suggest that strain M133^T^ can reduce nitrate to nitrogen.

Fe (III) or Mn (IV) reduction is an important anaerobic respiration pathway in acidobacteria. The outer membrane c-type cytochromes OmcA and MtrC (also known as OmcB) have been previously demonstrated to play an important role in the reduction of Fe (III) and Mn (III/IV) (Beliaev et al., 2001; Myers and Myers, 2001). *OmcA* and *MtrC* were detected in *T. aquaticum* MP-01^T^, *P. fermentans* P105^T^, and *Can.* S. usitatus Ellin6076. Both *T. aquaticum* MP-01^T^ (Losey et al., 2013) and *P. fermentans* P105^T^ (Kulichevskaya et al., 2014) have been reported to anaerobically reduce Fe (III) or Mn (IV). Fe (III) respiration in *Can* S. usitatus Ellin6076 needs to be confirmed using growth-based tests. The gene for MtrF, a homolog of MtrC, was detected in the genome of *G. ferrnentans* DSM 14018^T^, whereas no gene for OmcA was detected, although *G. ferrnentans* DSM 14018^T^ was confirmed to use Fe (III) as an electron acceptor (Coates et al., 1999).

Oxidized sulfur compounds are relatively low-efficient electron acceptors during respiration. Genes encoding assimilatory sulfate reductases were detected in 13 of the 21 acidobacterial genomes, and those encoding dissimilatory sulfate reductases were detected in two genomes (Supplementary Table 6). Genes encoding for sulfite reductase were detected in 16 genomes, of which only one (*H. foetida* DSM 6591^T^) was dissimilatory sulfite reductase (Supplementary Table 6). Only assimilatory sulfate and sulfite reduction genes were detected in strain M133 and *A. pedis* KCTC 12899^T^.

The bifunctional enzyme sulfhydrogenase was first reported in the anaerobic archaeon *Pyrococcus furiosus*, and this enzyme produces sulfide hydrogen from elemental sulfur or polysulfide under anaerobic conditions (Ma et al., 1993). Complete sulfhydrogenase genes *hydBGDA* (Pedroni et al., 1995) and accessory genes *hypCDEF* were also detected in the genome of strain M133. Therefore, sulfhydrogenase may function as a secondary anaerobic respiration pathway in this strain. High nutrition, especially tryptone or other protein derivatives, promotes hydrogen sulfide production. However, the actual substrate of sulfhydrogenase in strain M133 remains unclear. In addition, the amino acid sequence of the M133 sulfhydrogenase β subunit (homolog to sulfite reductase) was searched using NCBI BLAST, and both sulfite reductase and 4Fe-4S dicluster-containing proteins were retrieved. After manually checking the RASTtk re-annotated genomes in Kbase, approximately 31 genomes were confirmed to harbor complete sulfhydrogenase genes (αβγδ) (Supplementary Table 7). Phylogenetically, these sulfhydrogenase-containing strains belonged to approximately 10 bacterial phyla, including *Acidobacteria*, *Cyanobacteria*, “Chloroflexi,” “Deinococcus-Thermus,” “Nitrospinae,” *Proteobacteria* (αβγδ), *Bacteroidetes*, *Actinobacteria*, *Planctomycetes*, and *Verrucomicrobia*, and two archaeon phyla. Although hydrogenase and sulfite reductase were detected in *Firmicutes* (*Clostridium*), no sulfhydrogenase was found. Three archaeon strains from *Euryarchaeota* and *Thaumarchaeota*, including *Pyrococcus furiosus* DSM 3638, were also found to harbor complete sulfhydrogenase genes. This universally and randomly taxonomic distribution pattern suggests that HGT may have contributed to the dissemination of sulfhydrogenase. To examine the possibility of HGT, the G + C content of *hydBGDA* and the corresponding genome (where the *hydBGDA* were extracted) were analyzed. Results indicated that the G + C content of *hydBGDA* is highly variable among genomes (32.84–74.08 mol%), while the G + C content difference between *hydBGDA* and the whole genome is usually 2–4 mol% (Supplementary Table 7). This difference between a common ancestor and the individual genomes is a long-term evolutionary result. Therefore, the existence of sulfhydrogenase in strain M133 is likely the evolutionary remnant and not a result of recent HGT.

Several strains have been reported to undergo fermentative growth (*T. aquaticum* MP-01, *G. fermentans* DSM14018, *H. foetida* TMBS4, and *A. ailaaui* PMMR2) (Liesack et al., 1994; Coates et al., 1999; Losey et al., 2013; Myers and King, 2016). However, the function of the possible fermentation enzymes remains to be confirmed (Eichorst et al., 2018). Genes involved in fermentation, such as lactate dehydrogenase, fumarate reductase/succinate dehydrogenase, and phosphate acetyltransferase, were also detected in strain M133 and *A. pedis* KCTC 12899^T^; however, no fermentation growth was detected in the API 50 CH sheet.

### Acidobacteria–Animal Interaction

To date, species closely related to strain M133^T^ (>92% 16S rRNA gene identity) have seldom been detected in the environment. To explore these allied species, GenBank databases were blasted using the 16S rRNA gene sequences of strain M133^T^ and *A. pedis* KCTC 12899^T^. Only two operational taxonomic units (OTUs) (GU319135 and MT858037) were retrieved from the standard nucleotide database. SRA datasets for seawater and algae (Mei et al., 2019), sediment (Zhang et al., 2017; Ghate et al., 2021), coral and coral reefs (Liang et al., 2017; Pearman et al., 2019; Chen et al., 2021), and sponge (Baquiran and Conaco, 2018; Wu et al., 2018) were blasted, and the results indicated that strain M133^T^ allied reads were occasionally detected in the datasets of the Red Sea coral reef (PRJNA479721) and the Weizhou Island coral *P. lutea* (PRJNA786650). This occasional appearance of strain M133^T^ allied reads in high throughput sequencing datasets indicated that these bacteria do exist in related environments, but are usually not detected. Technically, DNA template preparation and PCR amplification can influence the outcome of certain reads. It is possible that the allied species exist in very low abundance in related habitats and/or cells are resistant to universal DNA extraction methods. Another reason may be the bias from the universal primer (Takahashi et al., 2014) and the barcode of indexed-primer (O’Donnell et al., 2016) used in PCR amplification. The exact cause of the low detection of strain M133^T^ allied species requires further study.

To date, the hosts or habitats of the allied species of strain M133^T^ have been limited to animals. Strain M133^T^ was isolated from the coral *P. lutea*, and five reads (100% identities to strain M133^T^) were detected in another coral *P. lutea* 1 year later (PRJNA786650). Interestingly, the bacterium clone A3M_UNP0_21 (GU319135, with 98% 16S rRNA sequence identity to strain M133^T^) was also detected in a coral (*Acropora eurystoma* in Israel) (Meron et al., 2011). Meanwhile, *A. pedis* KCTC 12899^T^ was isolated from the chiton *Acanthopleura japonica* in Japan (Fukunaga et al., 2008), and its closest bacterial clone OTU20 (MT858037, 100% identity) was detected in the gut of fish *Seriola rivoliana* in Mexico. Although the exact niches of the allied reads of strain M133^T^ (92–99% to strain M133^T^ or *A. pedis* KCTC 12899^T^) are unclear in the PVC plates deployed in the Red Sea coral reefs (Pearman et al., 2019), it is evident that benthic animal species could settle on similar autonomous reef monitoring structures (Leray and Knowlton, 2015), raising the possibility that allied reads were also from animals. In addition, some distant *Acanthopleuribacteraceae* reads also displayed an intimate association with coral. After three months of autotrophic incubation and weekly axenic washing, the abundance of *Acanthopleuribacteraceae* reads (85.9–91.7% identities to strain M133^T^, PRJNA787388) increased evidently in coral *P. lutea*, coinciding with the significant bacterial community composition shift (Supplementary Figures 3, 4). As the survivors of long-term autotrophy and repeated axenic wash, these distant reads should also be closely correlated.

In this study, it can be observed that strain M133^T^ and *A. pedis* KCTC 12899^T^ have evolved to associate with animals. Both strain M133^T^ and *A. pedis* KCTC 12899^T^ possess the ability to initiate and maintain symbiotic interactions with animals. Microbial motility and chemotaxis are pivotal for the onset and maintenance of symbiotic interactions (Raina et al., 2019). Moreover, both acidobacteria are mobile by using the flagella (Supplementary Figure 5; Fukunaga et al., 2008). Ankyrins, which was also detected in strain M133^T^ and *A. pedis* KCTC 12899^T^, can aid bacteria in evading the eukaryotic immune system (Jahn et al., 2019) in bacteria-animal symbiosis. For intracellular symbiotic bacteria, there is usually a dramatic genome reduction (McCutcheon and Moran, 2011; Jäckle et al., 2019), so strain M133^T^ and *A. pedis* KCTC 12899^T^ are not likely to form an intracellular symbiosis with animals. The discovery of OTU20 (MT858037) in the fish gut suggests that strain M133^T^ and *A. pedis* KCTC 12899^T^ may also exist in the animal gut environment. Sulfatases can digest the highly sulfated glycans in the gut, whereas the activation of sulfatases requires post-translational modification catalyzed by radical S-adenosyl-L-methionine proteins (Benjdia et al., 2007, 2011). Both genes encoding for sulfatase and radical S-adenosyl-L-methionine proteins were detected in the genomes of strain M133^T^ and *A. pedis* KCTC 12899^T^. The low oxygen and nitrate respiration capabilities of these bacteria further facilitate their survival and function in animal gut-like environments. The general metabolic pattern indicates that there is a positive selection resulting from long-term animal associations. Glycoside hydrolases related to plant polysaccharide degradation, such as alpha-L-rhamnosidase (GH106), endo-1,4-beta-xylanase (GH10), and alpha-galactosidase (GH27), are usually absent from strains M133^T^ and *A. pedis* KCTC 12899^T^. In contrast, animal disease-related virulence factors, such as collagenase (Harrington, 1996; Penttinen et al., 2016), astacin (Ricard-Blum and Vallet, 2016), adamalysin (Ricard-Blum and Vallet, 2016), subtilisin (Imamura et al., 2017; Łagowski et al., 2021), and thermolysin (Kong et al., 2015; Tsaplina et al., 2020) are present in strain M133^T^ and *A. pedis* KCTC 12899^T^.

Meanwhile, the allied species of strain M133^T^ may benefit animal hosts in dealing with environmental fluctuations. Several bacteria have been shown to protect their animal hosts from pathogens and heat stress, such as *Pseudoalteromonas* sp., which inhibits coral pathogens (*Vibrio* sp.) using antibiotics and peptidase (Offret et al., 2016; Richards et al., 2017; Rosado et al., 2019). *Ruegeria* sp. inhibits the coral pathogen *Vibrio coralliilyticus* (Miura et al., 2019), whereas *Muricauda* sp. protects coral endosymbionts from thermal stress by producing zeaxanthin (Motone et al., 2020). Although the exact products of BGCs are still unclear, the large secondary metabolite biosynthesis capacities of strain M133 and *A. pedis* KCTC 12899^T^ are still encouraging. According to antiSMASH known-cluster-blast, BGCs of strain M133 are very similar to the biosynthesis gene clusters of jerangolid A, oocydin A, nematophin, and microsclerodermin M. These compounds have been reported to have cytotoxic activities against fungi and gram-positive bacteria (Gerth et al., 1996; Strobel et al., 1999; Melikhova et al., 2016; Cai et al., 2017). Meanwhile, EPA accounts for 1% of the total fatty acids of the whole *P. lutea* holobionts; therefore, strain M133, an EPA-producer, may also benefit *P. lutea* holobionts in dealing with temperature and pressure variation (Valentine and Valentine, 2004).

### Taxonomy of the New Isolate

**FIGURE 2:**
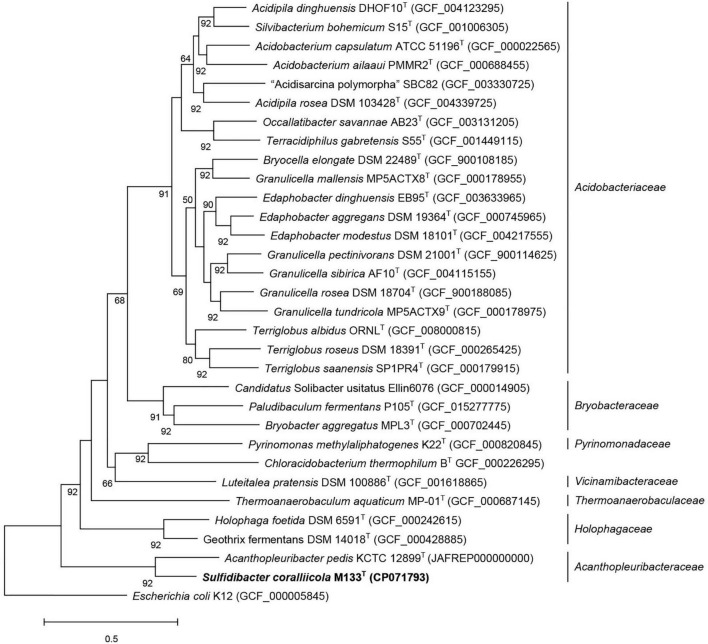
Phylogenomic tree inferred using UBCGs (concatenated alignment of 92 core genes) indicated strain M133^T^ belongs to the family *Acanthopleuribacteraceae*. Gene support indices (GSIs) is given at branching points, only above 50% is displayed. Bar, 0.5 substitution per position.

The closest taxonomic neighbor of strain M133^T^, based on the 16S rRNA gene sequence similarity, is *A. pedis* KCTC 12899^T^, they share 92.4% identity (recommended genus demarcation is 95%, Yarza et al., 2014). The average nucleotide identity between strains M133^T^ and KCTC 12899^T^ is 70% (recommended genus demarcation is 74%, Barco et al., 2020). The average amino-acid identity between strains M133^T^ and KCTC 12899^T^ is 63.97%, which is lower than the recommended genus demarcation of 68% (Konstantinidis and Tiedje, 2005). These indices indicated that strain M133^T^ and *A. pedis* KCTC 12899^T^ belong to different genera. Phylogenetic analysis based on the 16S rRNA gene sequences using maximum-likelihood, neighbor-joining, and maximum-parsimony algorithms (Supplementary Figures 4–6) and phylogenomic analysis using 92 concatenated sequence (Figure 2) indicated that strain M133^T^ forms a distinct branch beside *A. pedis* KCTC 12899^T^ in the family *Acanthopleuribacteraceae*.

Cells of strain M133^T^ are Gram-negative, non-spore-forming, motile, aerobic rods (Supplementary Figure 7). This strain can be distinguished from *A. pedis* KCTC 12899^T^ by the ability to reduce nitrate to nitrogen and the production of hydrogen sulfide. Chemotaxonomic features can also differentiate these two strains. The major cellular fatty acids (> 5%) of strain M133^T^ are isoC_15:0_ (35.6%), C_16:0_ (29.8%), C_15:0_ (8.1%), and C_20:5_ω3c (6.2%), while those of *A. pedis* KCTC 12899^T^ are C_16:0_ (29.6%), isoC_15:0_ (17.2%), isoC_17:0_ (12.4%), C_20:5_ω3c (12.2%), and C_16:0_N alcohol (5.8%) (Supplementary Table 5). The major polar lipids of strain M133^T^ are phosphatidylethanolamine, phosphatidylglycerol, and diphosphatidylglycerol, which are similar to those of *A. pedis* KCTC 12899^T^ (Supplementary Figure 8). Other features of strain M133^T^ are listed in Table 2 and the new taxon description.

**TABLE 2:** Characteristics distinguish strain M133^T^ from *Acanthopleuribacter pedis* KCTC 12899^T^.

Features	Strain M133^T^	*A. pedis* KCTC 12899^T^
</ltx:thead>Habitat	Sponge	Chiton
Cell size (μm)	0.9–1.5 × 2.3–5.3	0.7-1.0 × 2.4-4.7
Flagella	Single polar	Peritrichous
Growth temperature (°C)	15–37(25–30)	15–37(25–30)
Salinity (%, w/v)	0–10(0–3)	0.5–9(0-3)
Growth pH	4–10(7–8)	8–10(8)
Nitrate to nitrogen	+	–
Hydrogen sulfide production	+	–
Genome size (Mb)	11.79	10.85
Genome G + C content (mol%)	60.2	57.3
Coding sequences	7753	7575
COGs families	1925	1876
CAZymes families	63	56
Peptidases families	84	79
16S rRNA identity to M133^T^ (100%)	100	92.4
ANI to M133^T^ (%)	100	70
AAI to M133^T^ (%)	100	63.97
Major polar lipids	PE, PG, DPG	PE, PG, DPG
Major cellular fatty acids (>5%)	isoC_15:0_, C_16:0_, C_15:0_, C_20:5_ω3c	isoC_15:0_, C_16:0_, C_16:0_ N alcohol, isoC_17:0_, C_20:5_ω3c

*PE, phosphatidylethanolamine; PG, phosphatidylglycerol; DPG, diphosphatidylglycerol.*

In summary, phylogenomic, phylogenetic, chemotaxonomic and phenotypic differences to the closest type strain *A. pedis* KCTC 12899^T^ indicated that strain M133^T^ represents a new species in a new genus in the family *Acanthopleuribacteraceae*, for which *Sulfidibacter corallicola* gen. nov., sp. nov. is proposed.

### Description of *Sulfidibacter* gen. nov

*Sulfidibacter* (sul.fi.di.bac’ter. N.L. neut. n. *sulfidum*, sulfide; N.L. masc. n. *bacter*, rod; N. L. masc. n. *Sulfidibacter*, sulfide producing rod).

Cells are Gram-stain negative, non-spore-forming, motile, aerobic rods. Catalase and oxidase are positive. Nitrate reduction is positive. Major respiratory quinone are menaquinone 6 and 7 (MK6 & 7). Major cellular fatty acids are isoC_15:0_, C_16:0_, C_15:0_ and C_20:5_ω3c. Major polar lipids are phosphatidylethanolamine, phosphatidylglycerol, and diphosphatidylglycerol.

Type species is *Sulfidibacter corallicola.*

### Description of *Sulfidibacter corallicola* sp. nov

*Sulfidibacter corallicola* (co.ral.li′co.la. L. neut. n. *corallum*, coral; L. masc. suff. -*cola*, inhabitant dweller; N.L. n. *corallicola*, coral-dweller).

Cells have following features in addition to the genus description. Cells are usually 2.3–5.3 μm in length and 0.9–1.5 μm in width. Cells are motile by single polar flagellum. Colonies on marine agar are circular, smooth and yellow in color. H_2_S can be produced when incubated in high strength of marine agar or marine broth. Cells can grow at 15–37°C, pH4-10 under 0–10% NaCl (w/v). Starch, tween20, 40 and 60 are degraded. Nitrate is reduced to nitrogen. Arginine dihydrolase, urease, β-glucosidase, β-galactosidase and protease are positive in API 20NE test. Alkaline phosphatase, esterase C4, esterase C8, lipase C14, leucine arylamidase, valine arylamidase, cystine arylamidase, trypsin, chymotrypsin, acid phosphatase, naphthol-AS-BI-phosphohydrolase and N-acetyl-β-glucosaminidase are positive in API ZYM test. D-maltose, D-trehalose, D-cellobiose, gentiobiose, sucrose, turanose, D-salicin, N-acetyl-β-glucosamine, N-acetyl-β-galactosamine, N-acetyl neuraminic acid, D-glucose, D-fucose, L-fucose, inosine, D-arabitol, myo-inositol, glycerol, glucose-6-PO_4_, D-fructose-6-PO_4_, gelatin, L-aspartic acid, L-glutamic acid, L-histidine, L-pyroglutamic acid, L-serine, D-galacturonic acid, D-gluconic acid, D-glucuronic acid, glucuronamide, quinic acid, D-saccharic acid, D-lactic acid methyl ester, L-lactic acid, citric acid, α-keto-glutaric acid, D-malic acid, Tween40, γ-amino-butyric acid, β-hydroxy-butyric acid, α-keto-butyric acid, acetic acid and formic acid are oxidized in Biolog GenIII microplate. The cellular fatty acids also contain C_14:0_, isoC_17:0_, isoC_11:0_, C_14:1_ω7c, isoC_13:0_. The polar lipids also contain one phospholipid, five unidentified ninhydrin-positive lipids, and three unidentified polar lipids.

Type strain M133^T^ (=MCCC 1K03775^T^ = KCTC 72445^T^), was isolated from coral *Porites lutea*, China. Genome accession in GenBank is CP071793, and the DNA G + C content is 60.2 mol%.

## Conclusion

Species belonging to phylum *Acidobacteria* are ubiquitous among animals, yet their ecological role remains unclear. In this study, comparative genomic and high-throughput sequencing analyses indicated that *Acanthopleuribacteraceae* is a distinct branch of the phylum *Acidobacteria*, features a large genome that harbors genes associated with secondary metabolic production, tolerance to oxygen fluctuation, and intimate animal association. Phylogenetic, phenotypic, and taxonomic analysis indicated that strain M133^T^ represents a new species in a new genus in the family *Acanthopleuribacteraceae*. To date, strain M133^T^ and *A. pedis* KCTC 12899^T^ are the only two acidobacterial isolates obtained from animals, which are not sufficient to disclose the roles of acidobacteria in animals. Therefore, pure cultures of acidobacteria from animals are urgently needed. For strain M133^T^ and *A. pedis* KCTC 12899^T^, their exact ecological niches, community volume, and the form and material they interact with animals need further study.

## Data Availability Statement

The datasets presented in this study can be found in online repositories. The names of the repository/repositories and accession number(s) can be found below: https://www.ncbi.nlm.nih.gov/genbank/, CP071793; https://www.ncbi.nlm.nih.gov/genbank/, JAFREP000000000.

## Author Contributions

GW and KY derived the idea for the whole project. YL and JFL performed the experiments. GW performed the bioinformatics analysis and wrote the manuscript. BC and WH provided the samples. BC, HS, and JYL joined discussion of the manuscript. All authors contributed to the article and approved the submitted version.

## Conflict of Interest

The authors declare that the research was conducted in the absence of any commercial or financial relationships that could be construed as a potential conflict of interest.

## Publisher’s Note

All claims expressed in this article are solely those of the authors and do not necessarily represent those of their affiliated organizations, or those of the publisher, the editors and the reviewers. Any product that may be evaluated in this article, or claim that may be made by its manufacturer, is not guaranteed or endorsed by the publisher.

## References

[B1] Anders S. Pyl P. T. Huber W. (2015). HTSeq–a Python framework to work with high-throughput sequencing data. *Bioinformatics* 31 166–169. 10.1093/bioinformatics/btu638 25260700 PMC4287950

[B2] Ardui S. Ameur A. Vermeesch J. R. Hestand M. S. (2018). Single molecule real-time (SMRT) sequencing comes of age: applications and utilities for medical diagnostics. *Nucleic Acids Res.* 46 2159–2168. 10.1093/nar/gky066 29401301 PMC5861413

[B3] Arkin A. P. Cottingham R. W. Henry C. S. Harris N. L. Stevens R. L. Maslov S. (2018). KBase: the United States Department of Energy Systems Biology Knowledgebase. *Nat. Biotechnol.* 36 566–569. 10.1038/nbt.4163 29979655 PMC6870991

[B4] Arndt D. Grant J. R. Marcu A. Sajed T. Pon A. Liang Y. (2016). PHASTER: a better, faster version of the PHAST phage search tool. *Nucleic Acids Res.* 44 W16–W21. 10.1093/nar/gkw387 27141966 PMC4987931

[B5] Aziz R. K. Bartels D. Best A. A. DeJongh M. Disz T. Edwards R. A. (2008). The RAST Server: rapid annotations using subsystems technology. *BMC Genomics* 9:75. 10.1186/1471-2164-9-75 18261238 PMC2265698

[B6] Bankevich A. Nurk S. Antipov D. Gurevich A. A. Dvorkin M. Kulikov A. S. (2012). SPAdes: a new genome assembly algorithm and its applications to single-cell sequencing. *J. Comput. Biol.* 19 455–477. 10.1089/cmb.2012.0021 22506599 PMC3342519

[B7] Baquiran J. Conaco C. (2018). Sponge-microbe partnerships are stable under eutrophication pressure from mariculture. *Mar. Pollut Bull.* 136 125–134. 10.1016/j.marpolbul.2018.09.011 30509793

[B8] Barco R. A. Garrity G. M. Scott J. J. Amend J. P. Nealson K. H. Emerson D. (2020). A Genus Definition for Bacteria and Archaea Based on a Standard Genome Relatedness Index. *mBio* 11 e02475–19. 10.1128/mBio.02475-19 31937639 PMC6960282

[B9] Barns S. M. Takala S. L. Kuske C. R. (1999). Wide distribution and diversity of members of the bacterial kingdom *Acidobacterium* in the environment. *Appl. Environ. Microbiol.* 65 1731–1737. 10.1128/AEM.65.4.1731-1737.1999 10103274 PMC91244

[B10] Beliaev A. S. Saffarini D. A. McLaughlin J. L. Hunnicutt D. (2001). MtrC, an outer membrane decahaem c cytochrome required for metal reduction in *Shewanella putrefaciens* MR-1. *Mol. Microbiol.* 39 722–730. 10.1046/j.1365-2958.2001.02257.x 11169112

[B11] Belova S. E. Ravin N. V. Pankratov T. A. Rakitin A. L. Ivanova A. A. Beletsky A. V. (2018). Hydrolytic Capabilities as a Key to Environmental Success: chitinolytic and Cellulolytic *Acidobacteria* From Acidic Sub-arctic Soils and Boreal Peatlands. *Front. Microbiol.* 9:2775. 10.3389/fmicb.2018.02775 30510549 PMC6252331

[B12] Benjdia A. Leprince J. Guillot A. Vaudry H. Rabot S. Berteau O. (2007). Anaerobic sulfatase-maturating enzymes: radical SAM enzymes able to catalyze in vitro sulfatase post-translational modification. *J. Am. Chem. Soc.* 129 3462–3463. 10.1021/ja067175e 17335281

[B13] Benjdia A. Martens E. C. Gordon J. I. Berteau O. (2011). Sulfatases and a radical S-adenosyl-L-methionine (AdoMet) enzyme are key for mucosal foraging and fitness of the prominent human gut symbiont, *Bacteroides thetaiotaomicron*. *J. Biol. Chem.* 286 25973–25982. 10.1074/jbc.M111.228841 21507958 PMC3138274

[B14] Bertelli C. Laird M. R. Williams K. P. Simon Fraser University Research Computing Group, Lau B. Y. Hoad G. (2017). IslandViewer 4: expanded prediction of genomic islands for larger-scale datasets. *Nucleic Acids Res.* 45 W30–W35. 10.1093/nar/gkx343 28472413 PMC5570257

[B15] Besemer J. Lomsadze A. Borodovsky M. (2001). GeneMarkS: a self-training method for prediction of gene starts in microbial genomes. Implications for finding sequence motifs in regulatory regions. *Nucleic Acids Res.* 29 2607–2618. 10.1093/nar/29.12.2607 11410670 PMC55746

[B16] Blin K. Shaw S. Kloosterman A. M. Charlop-Powers Z. van Wezel G. P. Medema M. H. (2021). antiSMASH 6.0: improving cluster detection and comparison capabilities. *Nucleic Acids Res.* 49 W29–W35. 10.1093/nar/gkab335 33978755 PMC8262755

[B17] Boutanaev A. M. Osbourn A. E. (2018). Multigenome analysis implicates miniature inverted-repeat transposable elements (MITEs) in metabolic diversification in eudicots. *Proc. Natl. Acad. Sci. U. S. A.* 115 E6650–E6658. 10.1073/pnas.1721318115 29941591 PMC6048515

[B18] Bush P. K. (2020). Accurate, automatic annotation of peptidases with hotpep-protease. *Green Chem. Eng*. 1 124–130. 10.1016/j.gce.2020.11.008

[B19] Cai X. Challinor V. L. Zhao L. Reimer D. Adihou H. Grün P. (2017). Biosynthesis of the Antibiotic Nematophin and Its Elongated Derivatives in Entomopathogenic Bacteria. *Org. Lett.* 19 806–809. 10.1021/acs.orglett.6b03796 28134534

[B20] Challacombe J. Kuske C. (2012). Mobile genetic elements in the bacterial phylum *Acidobacteria*. *Mob. Genet. Elements* 2 179–183. 10.4161/mge.21943 23087842 PMC3469429

[B21] Challacombe J. F. Eichorst S. A. Hauser L. Land M. Xie G. Kuske C. R. (2011). Biological consequences of ancient gene acquisition and duplication in the large genome of *Candidatus* Solibacter usitatus Ellin6076. *PLoS One* 6:e24882. 10.1371/journal.pone.0024882 21949776 PMC3174227

[B22] Chanal A. Chapon V. Benzerara K. Barakat M. Christen R. Achouak W. (2006). The desert of Tataouine: an extreme environment that hosts a wide diversity of microorganisms and radiotolerant bacteria. *Environ. Microbiol.* 8 514–525. 10.1111/j.1462-2920.2005.00921.x 16478457

[B23] Chen B. Yu K. Liao Z. Yu X. Qin Z. Liang J. (2021). Microbiome community and complexity indicate environmental gradient acclimatisation and potential microbial interaction of endemic coral holobionts in the South China Sea. *Sci. Total Environ.* 765:142690. 10.1016/j.scitotenv.2020.142690 33071127

[B24] Coates J. D. Ellis D. J. Gaw C. V. Lovley D. R. (1999). *Geothrix fermentans* gen. nov., sp. nov., a novel Fe(III)-reducing bacterium from a hydrocarbon-contaminated aquifer. *Int. J. Syst. Bacteriol.* 49 1615–1622. 10.1099/00207713-49-4-1615 10555343

[B25] Crowe M. A. Power J. F. Morgan X. C. Dunfield P. F. Lagutin K. Rijpstra W. (2014). *Pyrinomonas methylaliphatogenes* gen. nov., sp. nov., a novel group 4 thermophilic member of the phylum Acidobacteria from geothermal soils. *Int. J. Syst. Evol. Microbiol.* 64 220–227. 10.1099/ijs.0.055079-0 24048862

[B26] de Castro V. H. Schroeder L. F. Quirino B. F. Kruger R. H. Barreto C. C. (2013). Acidobacteria from oligotrophic soil from the Cerrado can grow in a wide range of carbon source concentrations. *Can. J. Microbiol*. 59 746–753. 10.1139/cjm-2013-0331 24206357

[B27] de Chaves M. G. Silva G. Rossetto R. Edwards R. A. Tsai S. M. Navarrete A. A. (2019). *Acidobacteria* Subgroups and Their Metabolic Potential for Carbon Degradation in Sugarcane Soil Amended With Vinasse and Nitrogen Fertilizers. *Front. Microbiol.* 10:1680. 10.3389/fmicb.2019.01680 31417506 PMC6682628

[B28] Dedysh S. N. Kulichevskaya I. S. Serkebaeva Y. M. Mityaeva M. A. Sorokin V. V. Suzina N. E. (2012). *Bryocella elongata* gen. nov., sp. nov., a member of subdivision 1 of the *Acidobacteria* isolated from a methanotrophic enrichment culture, and emended description of *Edaphobacter aggregans* Koch 2008. *Int. J. Syst. Evol. Microbiol.* 62 654–664. 10.1099/ijs.0.031898-0 21551329

[B29] Eichorst S. A. Breznak J. A. Schmidt T. M. (2007). Isolation and characterization of soil bacteria that define *Terriglobus* gen. nov., in the phylum *Acidobacteria*. *Appl. Environ. Microbiol.* 73 2708–2717. 10.1128/AEM.02140-06 17293520 PMC1855589

[B30] Eichorst S. A. Trojan D. Roux S. Herbold C. Rattei T. Woebken D. (2018). Genomic insights into the Acidobacteria reveal strategies for their success in terrestrial environments. *Environ. Microbiol.* 20 1041–1063. 10.1111/1462-2920.14043 29327410 PMC5900883

[B31] Falagán C. Foesel B. Johnson B. (2017). *Acidicapsa ferrireducens* sp. nov., *Acidicapsa acidiphila* sp. nov., and *Granulicella acidiphila* sp. nov.: novel acidobacteria isolated from metal-rich acidic waters. *Extremophiles* 21 459–469. 10.1007/s00792-017-0916-4 28229259

[B32] Felsenstein J. (1981). Evolutionary trees from DNA sequences: a maximum likelihood approach. *J. Mol. Evol.* 17 368–376. 10.1007/BF01734359 7288891

[B33] Frost L. S. Leplae R. Summers A. O. Toussaint A. (2005). Mobile genetic elements: the agents of open source evolution. *Nat. Rev. Microbiol.* 3 722–732. 10.1038/nrmicro1235 16138100

[B34] Fukunaga Y. Kurahashi M. Yanagi K. Yokota A. Harayama S. (2008). *Acanthopleuribacter pedis* gen. nov., sp. nov., a marine bacterium isolated from a chiton, and description of *Acanthopleuribacteraceae* fam. nov., *Acanthopleuribacterales* ord. nov., *Holophagaceae* fam. nov., *Holophagales* ord. nov. and *Holophagae* classis nov. in the phylum ‘Acidobacteria’. *Int. J. Syst. Evol. Microbiol.* 58 2597–2601. 10.1099/ijs.0.65589-0 18984699

[B35] Gallagher K. A. Jensen P. R. (2015). Genomic insights into the evolution of hybrid isoprenoid biosynthetic gene clusters in the MAR4 marine streptomycete clade. *BMC Genomics* 16:960. 10.1186/s12864-015-2110-3 26578069 PMC4650096

[B36] Gerth K. Washausen P. Hofle G. Irschik H. Reichenbach H. (1996). The jerangolids: a family of new antifungal compounds from *Sorangium cellulosum* (Myxobacteria). Production, physico-chemical and biological properties of jerangolid A. *J. Antibiot.* 49 71–75. 10.7164/antibiotics.49.71 8609090

[B37] Ghate S. D. Shastry R. P. Arun A. B. Rekha P. D. (2021). Unraveling the bacterial community composition across aquatic sediments in the Southwestern coast of India by employing high-throughput 16S rRNA gene sequencing. *Reg. Stud. Mar. Sci.* 46:101890. 10.1016/j.rsma.2021.101890

[B38] Gonçalves O. S. Santana M. F. (2021). The coexistence of monopartite integrative and conjugative elements in the genomes of Acidobacteria. *Gene* 777:145476. 10.1016/j.gene.2021.145476 33549716

[B39] Harrington D. J. (1996). Bacterial collagenases and collagen-degrading enzymes and their potential role in human disease. *Infect. Immun.* 64 1885–1891. 10.1128/iai.64.6.1885-1891.1996 8675283 PMC174012

[B40] Hausmann B. Pelikan C. Herbold C. W. Köstlbacher S. Albertsen M. Eichorst S. A. (2018). Peatland *Acidobacteria* with a dissimilatory sulfur metabolism. *ISME J.* 12 1729–1742. 10.1038/s41396-018-0077-1 29476143 PMC6018796

[B41] Imamura T. Murakami Y. Nitta H. (2017). *Aeromonas sobria* serine protease (ASP): a subtilisin family endopeptidase with multiple virulence activities. *Biol. Chem.* 398 1055–1068. 10.1515/hsz-2016-0344 28432839

[B42] Ivanova A. A. Zhelezova A. D. Chernov T. I. Dedysh S. N. (2020). Linking ecology and systematics of acidobacteria: distinct habitat preferences of the *Acidobacteriia* and *Blastocatellia* in tundra soils. *PLoS One* 15:e0230157. 10.1371/journal.pone.0230157 32182280 PMC7077872

[B43] Jäckle O. Seah B. Tietjen M. Leisch N. Liebeke M. Kleiner M. (2019). Chemosynthetic symbiont with a drastically reduced genome serves as primary energy storage in the marine flatworm Paracatenula. *Proc. Natl. Acad. Sci. U. S. A.* 116 8505–8514. 10.1073/pnas.1818995116 30962361 PMC6486704

[B44] Jahn M. T. Arkhipova K. Markert S. M. Stigloher C. Lachnit T. Pita L. (2019). A Phage Protein Aids Bacterial Symbionts in Eukaryote Immune Evasion. *Cell Host Microbe* 26 542–550.e5. 10.1016/j.chom.2019.08.019 31561965

[B45] Kalam S. Basu A. Ahmad I. Sayyed R. Z. El-Enshasy H. A. Dailin D. J. (2020). Recent Understanding of Soil Acidobacteria and Their Ecological Significance: a Critical Review. *Front. Microbiol.* 11:580024. 10.3389/fmicb.2020.580024 33193209 PMC7661733

[B46] Kanehisa M. Goto S. Kawashima S. Okuno Y. Hattori M. (2004). The KEGG resource for deciphering the genome. *Nucleic Acids Res.* 32 D277–D280. 10.1093/nar/gkh063 14681412 PMC308797

[B47] Kielak A. M. Cipriano M. A. Kuramae E. E. (2016). *Acidobacteria* strains from subdivision 1 act as plant growth-promoting bacteria. *Arch. Microbiol.* 198 987–993. 10.1007/s00203-016-1260-2 27339258 PMC5080364

[B48] Kim D. Park S. Chun J. (2021). Introducing EzAAI: a pipeline for high throughput calculations of prokaryotic average amino acid identity. *J. Microbiol*. 59 476–480. 10.1007/s12275-021-1154-0 33907973

[B49] Kishimoto N. Kosako Y. Tano T. (1991). *Acidobacterium capsulatum* gen. nov., sp. nov.: an acidophilic chemoorganotrophic bacterium containing menaquinone from acidic mineral environment. *Curr. Microbiol.* 22 1–7. 10.1007/BF02106205 23835745

[B50] Kong L. Lu A. Guan J. Yang B. Li M. Hillyer J. F. (2015). Thermolysin damages animal life through degradation of plasma proteins enhanced by rapid cleavage of serpins and activation of proteases. *Arch. Insect Biochem. Physiol.* 88 64–84. 10.1002/arch.21178 25042057

[B51] Konstantinidis K. T. Tiedje J. M. (2005). Towards a genome-based taxonomy for prokaryotes. *J. Bacteriol.* 187 6258–6264. 10.1128/JB.187.18.6258-6264.2005 16159757 PMC1236649

[B52] Krzywinski M. Schein J. Birol I. Connors J. Gascoyne R. Horsman D. (2009). Circos: an information aesthetic for comparative genomics. *Genome Res.* 19 1639–1645. 10.1101/gr.092759.109 19541911 PMC2752132

[B53] Kulichevskaya I. S. Suzina N. E. Liesack W. Dedysh S. N. (2010). Bryobacter aggregatus gen. nov., sp. nov., a peat-inhabiting, aerobic chemo-organotroph from subdivision 3 of the *Acidobacteria*. *Int. J. Syst. Evol. Microbiol.* 60 301–306. 10.1099/ijs.0.013250-0 19651730

[B54] Kulichevskaya I. S. Suzina N. E. Rijpstra W. Damsté J. Dedysh S. N. (2014). Paludibaculum fermentans gen. nov., sp. nov., a facultative anaerobe capable of dissimilatory iron reduction from subdivision 3 of the *Acidobacteria*. *Int. J. Syst. Evol. Microbiol.* 64 2857–2864. 10.1099/ijs.0.066175-0 24867171

[B55] Kumar S. Stecher G. Tamura K. (2016). MEGA7: molecular Evolutionary Genetics Analysis version 7.0 for bigger datasets. *Mol. Biol. Evol.* 33 1870–1874. 10.1093/molbev/msw054 27004904 PMC8210823

[B56] Łagowski D. Gnat S. Nowakiewicz A. Osińska M. (2021). Assessment of the subtilisin gene profile in *Trichophyton verrucosum* isolated from human and animal dermatophytoses in two-stage multiplex PCR. *J. Appl. Microbiol.* 131 300–306. 10.1111/jam.14942 33245823

[B57] Langmead B. Salzberg S. L. (2012). Fast gapped-read alignment with Bowtie 2. *Nat. Methods* 9 357–359. 10.1038/nmeth.1923 22388286 PMC3322381

[B58] Laport M. S. Pinheiro U. Rachid C. (2019). Freshwater Sponge *Tubella variabilis* Presents Richer Microbiota Than Marine Sponge Species. *Front. Microbiol.* 10:2799. 10.3389/fmicb.2019.02799 31849922 PMC6902092

[B59] Leray M. Knowlton N. (2015). DNA barcoding and metabarcoding of standardized samples reveal patterns of marine benthic diversity. *Proc. Natl. Acad. Sci. U. S. A.* 112 2076–2081. 10.1073/pnas.1424997112 25646458 PMC4343139

[B60] Liang J. Yu K. Wang Y. Huang X. Huang W. Qin Z. (2017). Distinct bacterial communities associated with massive and branching scleractinian corals and potential linkages to coral susceptibility to thermal or cold stress. *Front. Microbiol.* 8:979. 10.3389/fmicb.2017.00979 28642738 PMC5462945

[B61] Liesack W. Bak F. Kreft J. U. Stackebrandt E. (1994). *Holophaga foetida* gen. nov., sp. nov., a new, homoacetogenic bacterium degrading methoxylated aromatic compounds. *Arch. Microbiol.* 162 85–90. 10.1007/BF00264378 8085918

[B62] Lim H. J. Lee E. H. Yoon Y. Chua B. Son A. (2016). Portable lysis apparatus for rapid single-step DNA extraction of *Bacillus subtilis*. *J. Appl. Microbiol.* 120 379–387. 10.1111/jam.13011 26606545

[B63] Lin S. H. Liao Y. C. (2013). CISA: contig integrator for sequence assembly of bacterial genomes. *PLoS One* 8:e60843. 10.1371/journal.pone.0060843 23556006 PMC3610655

[B64] Lladó S. Benada O. Cajthaml T. Baldrian P. García-Fraile P. (2016). *Silvibacterium bohemicum* gen. nov. sp. nov., an acidobacterium isolated from coniferous soil in the Bohemian Forest National Park. *Syst. Appl. Microbiol.* 39 14–19. 10.1016/j.syapm.2015.12.005 26774420

[B65] Lopez J. V. (2003). Naturally mosaic operons for secondary metabolite biosynthesis: variability and putative horizontal transfer of discrete catalytic domains of the epothilone polyketide synthase locus. *Mol. Genet. Genomics* 270 420–431. 10.1007/s00438-003-0937-9 14595556

[B66] Losey N. A. Stevenson B. S. Busse H. J. Damsté J. Rijpstra W. Rudd S. (2013). *Thermoanaerobaculum aquaticum* gen. nov., sp. nov., the first cultivated member of Acidobacteria subdivision 23, isolated from a hot spring. *Int. J. Syst. Evol. Microbiol.* 63 4149–4157. 10.1099/ijs.0.051425-0 23771620

[B67] Luo R. Liu B. Xie Y. Li Z. Huang W. Yuan J. (2012). SOAPdenovo2: an empirically improved memory-efficient short-read de novo assembler. *Gigascience* 1:18. 10.1186/2047-217X-1-18 23587118 PMC3626529

[B68] Ma K. Schicho R. N. Kelly R. M. Adams M. W. (1993). Hydrogenase of the hyperthermophile *Pyrococcus furiosus* is an elemental sulfur reductase or sulfhydrogenase: evidence for a sulfur-reducing hydrogenase ancestor. *Proc. Natl. Acad. Sci. U. S. A.* 90 5341–5344. 10.1073/pnas.90.11.5341 8389482 PMC46712

[B69] McClure R. Balasubramanian D. Sun Y. Bobrovskyy M. Sumby P. Genco C. A. (2013). Computational analysis of bacterial RNA-Seq data. *Nucleic Acids Res.* 41:e140. 10.1093/nar/gkt444 23716638 PMC3737546

[B70] McCutcheon J. P. Moran N. A. (2011). Extreme genome reduction in symbiotic bacteria. *Nat. Rev. Microbiol.* 10 13–26. 10.1038/nrmicro2670 22064560

[B71] McFall-Ngai M. Hadfield M. G. Bosch T. C. Carey H. V. Domazet-Lošo T. Douglas A. E. (2013). Animals in a bacterial world, a new imperative for the life sciences. *Proc. Natl. Acad. Sci. U. S. A.* 110 3229–3236. 10.1073/pnas.1218525110 23391737 PMC3587249

[B72] Medema M. H. Kottmann R. Yilmaz P. Cummings M. Biggins J. B. Blin K. (2015). Minimum Information about a Biosynthetic Gene cluster. *Nat. Chem. Biol.* 11 625–631. 10.1038/nchembio.1890 26284661 PMC5714517

[B73] Meekrathok P. Bürger M. Porfetye A. T. Kumsaoad S. Aunkham A. Vetter I. R. (2021). Structural basis of chitin utilization by a GH20 β-N-acetylglucosaminidase from *Vibrio campbellii* strain ATCC BAA-1116. *Acta Crystallogr. D Struct. Biol.* 77 674–689. 10.1107/S2059798321002771 33950022 PMC8098473

[B74] Mei X. Wu C. Zhao J. Yan T. Jiang P. (2019). Community Structure of Bacteria Associated With Drifting Sargassum horneri, the Causative Species of Golden Tide in the Yellow Sea. *Front. Microbiol.* 10:1192. 10.3389/fmicb.2019.01192 31191503 PMC6546727

[B75] Melikhova E. Y. Pullin R. D. Winter C. Donohoe T. J. (2016). Dehydromicrosclerodermin B and Microsclerodermin J: total Synthesis and Structural Revision. *Angew. Chem. Int.* Ed. Engl. 55 9753–9757. 10.1002/anie.201604764 27418203 PMC5132153

[B76] Meron D. Atias E. Iasur Kruh L. Elifantz H. Minz D. Fine M. (2011). The impact of reduced pH on the microbial community of the coral *Acropora eurystoma*. *ISME J.* 5 51–60. 10.1038/ismej.2010.102 20668489 PMC3105665

[B77] Miura N. Motone K. Takagi T. Aburaya S. Watanabe S. Aoki W. (2019). Ruegeria sp. Strains Isolated from the Reef-Building Coral *Galaxea fascicularis* Inhibit Growth of the Temperature-Dependent Pathogen *Vibrio coralliilyticus*. *Mar. Biotechnol.* 21 1–8. 10.1007/s10126-018-9853-1 30194504

[B78] Mohr K. I. Wolf C. Nübel U. Szafrańska A. K. Steglich M. Hennessen F. (2018). A polyphasic approach leads to seven new species of the cellulose-decomposing genus *Sorangium*, *Sorangium ambruticinum* sp. nov., *Sorangium arenae* sp. nov., *Sorangium bulgaricum* sp. nov., *Sorangium dawidii* sp. nov., *Sorangium kenyense* sp. nov., *Sorangium orientale* sp. nov. and *Sorangium reichenbachii* sp. nov. *Int. J. Syst. Evol. Microbiol.* 68 3576–3586. 10.1099/ijsem.0.003034 30234476

[B79] Morozkina E. V. Zvyagilskaya R. A. (2007). Nitrate reductases: structure, functions, and effect of stress factors. *Biochemistry* 72 1151–1160. 10.1134/s0006297907100124 18021072

[B80] Morris R. L. Schmidt T. M. (2013). Shallow breathing: bacterial life at low O_2_. *Nat. Rev. Microbiol.* 11 205–212. 10.1038/nrmicro2970 23411864 PMC3969821

[B81] Motone K. Takagi T. Aburaya S. Miura N. Aoki W. Ueda M. (2020). A Zeaxanthin-Producing Bacterium Isolated from the Algal Phycosphere Protects Coral Endosymbionts from Environmental Stress. *mBio* 11 e01019–19. 10.1128/mBio.01019-19 31964724 PMC6974559

[B82] Myers J. M. Myers C. R. (2001). Role for outer membrane cytochromes OmcA and OmcB of *Shewanella putrefaciens* MR-1 in reduction of manganese dioxide. *Appl. Environ. Microbiol.* 67 260–269. 10.1128/AEM.67.1.260-269.2001 11133454 PMC92560

[B83] Myers M. R. King G. M. (2016). Isolation and characterization of *Acidobacterium ailaaui* sp. nov., a novel member of *Acidobacteria* subdivision 1, from a geothermally heated Hawaiian microbial mat. *Int. J. Syst. Evol. Microbiol.* 66 5328–5335. 10.1099/ijsem.0.001516 27692038

[B84] Na S. I. Kim Y. O. Yoon S. H. Ha S. M. Baek I. Chun J. (2018). UBCG: up-to-date bacterial core gene set and pipeline for phylogenomic tree reconstruction. *J. Microbiol.* 56 280–285. 10.1007/s12275-018-8014-6 29492869

[B85] Naumoff D. G. (2016). GH10 Family of Glycoside Hydrolases: structure and Evolutionary Connections. *Mol. Biol.* 50 151–160. 10.7868/S0026898415060208 27028821

[B86] Naumoff D. G. Dedysh S. N. (2012). Lateral gene transfer between the *Bacteroidetes* and *Acidobacteria*: the case of α-L-rhamnosidases. *FEBS Lett.* 586 3843–3851. 10.1016/j.febslet.2012.09.005 23022563

[B87] Navarrete A. A. Venturini A. M. Meyer K. M. Klein A. M. Tiedje J. M. Bohannan B. J. (2015). Differential Response of Acidobacteria Subgroups to Forest-to-Pasture Conversion and Their Biogeographic Patterns in the Western Brazilian Amazon. *Front. Microbiol.* 6:1443. 10.3389/fmicb.2015.01443 26733981 PMC4686610

[B88] O’Connor-Sánchez A. Rivera-Domínguez A. J. Santos-Briones C. López-Aguiar L. K. Peña-Ramírez Y. J. Prieto-Davo A. (2014). *Acidobacteria* appear to dominate the microbiome of two sympatric Caribbean Sponges and one Zoanthid. *Biol. Res.* 47:67. 10.1186/0717-6287-47-67 25723107 PMC4335776

[B89] O’Donnell J. L. Kelly R. P. Lowell N. C. Port J. A. (2016). Indexed PCR Primers Induce Template-Specific Bias in Large-Scale DNA Sequencing Studies. *PLoS One* 11:e0148698. 10.1371/journal.pone.0148698 26950069 PMC4780811

[B90] Offret C. Desriac F. Le Chevalier P. Mounier J. Jégou C. Fleury Y. (2016). Spotlight on Antimicrobial Metabolites from the Marine Bacteria *Pseudoalteromonas*: chemodiversity and Ecological Significance. *Mar. Drugs* 14:129. 10.3390/md14070129 27399731 PMC4962019

[B91] Okamura K. Kawai A. Yamada T. Hiraishi A. (2011). Acidipila rosea gen. nov., sp. nov., an acidophilic chemoorganotrophic bacterium belonging to the phylum *Acidobacteria*. *FEMS Microbiol. Lett.* 317 138–142. 10.1111/j.1574-6968.2011.02224.x 21255071

[B92] Pankratov T. A. Dedysh S. N. (2010). *Granulicella paludicola* gen. nov., sp. nov., *Granulicella pectinivorans* sp. nov., *Granulicella aggregans* sp. nov. and *Granulicella rosea* sp. nov., acidophilic, polymer-degrading acidobacteria from *Sphagnum* peat bogs. *Int. J. Syst. Evol. Microbiol.* 60 2951–2959. 10.1099/ijs.0.021824-0 20118293

[B93] Pearman J. K. Aylagas E. Voolstra C. R. Anlauf H. Villalobos R. Carvalho S. (2019). Disentangling the complex microbial community of coral reefs using standardized Autonomous Reef Monitoring Structures (ARMS). *Mol. Ecol.* 28 3496–3507. 10.1111/mec.15167 31281998 PMC6851789

[B94] Pedroni P. Della Volpe A. Galli G. Mura G. M. Pratesi C. Grandi G. (1995). Characterization of the locus encoding the [Ni-Fe] sulfhydrogenase from the archaeon *Pyrococcus furiosus*: evidence for a relationship to bacterial sulfite reductases. *Microbiology* 141 449–458. 10.1099/13500872-141-2-449 7704275

[B95] Penttinen R. Kinnula H. Lipponen A. Bamford J. K. H. Sundberg L. (2016). High Nutrient Concentration Can Induce Virulence Factor Expression and Cause Higher Virulence in an Environmentally Transmitted Pathogen. *Microb. Ecol.* 72 955–964. 10.1007/s00248-016-0781-1 27193154

[B96] Perreau J. Moran N. A. (2022). Genetic innovations in animal-microbe symbioses. *Nat. Rev. Genet.* 23 23–39. 10.1038/s41576-021-00395-z 34389828 PMC8832400

[B97] Quaiser A. Ochsenreiter T. Lanz C. Schuster S. C. Treusch A. H. Eck J. (2003). Acidobacteria form a coherent but highly diverse group within the bacterial domain: evidence from environmental genomics. *Mol. Microbiol.* 50 563–575. 10.1046/j.1365-2958.2003.03707.x 14617179

[B98] Raina J. B. Fernandez V. Lambert B. Stocker R. Seymour J. R. (2019). The role of microbial motility and chemotaxis in symbiosis. *Nat. Rev. Microbiol.* 17 284–294. 10.1038/s41579-019-0182-9 30923350

[B99] Ricard-Blum S. Vallet S. D. (2016). Proteases decode the extracellular matrix cryptome. *Biochimie* 122 300–313. 10.1016/j.biochi.2015.09.016 26382969

[B100] Richards G. P. Watson M. A. Needleman D. S. Uknalis J. Boyd E. F. Fay J. P. (2017). Mechanisms for *Pseudoalteromonas piscicida*-Induced Killing of *Vibrios* and Other Bacterial Pathogens. *Appl. Environ. Microbiol.* 83 e00175–17. 10.1128/AEM.00175-17 28363962 PMC5440704

[B101] Robinson M. D. McCarthy D. J. Smyth G. K. (2010). edgeR: a Bioconductor package for differential expression analysis of digital gene expression data. *Bioinformatics* 26 139–140. 10.1093/bioinformatics/btp616 19910308 PMC2796818

[B102] Rosado P. M. Leite D. Duarte G. Chaloub R. M. Jospin G. Nunes da Rocha U. (2019). Marine probiotics: increasing coral resistance to bleaching through microbiome manipulation. *ISME J.* 13 921–936. 10.1038/s41396-018-0323-6 30518818 PMC6461899

[B103] Saitou N. Nei M. (1987). The neighbor-joining method: a new method for reconstructing phylogenetic trees. *Mol. Biol. Evol.* 4 406–425. 10.1093/oxfordjournals.molbev.a040454 3447015

[B104] Shulse C. N. Allen E. E. (2011). Widespread occurrence of secondary lipid biosynthesis potential in microbial lineages. *PLoS One* 6:e20146. 10.1371/journal.pone.0020146 21629834 PMC3098273

[B105] Simpson J. T. Wong K. Jackman S. D. Schein J. E. Jones S. J. Birol I. (2009). ABySS: a parallel assembler for short read sequence data. *Genome Res.* 19 1117–1123. 10.1101/gr.089532.108 19251739 PMC2694472

[B106] Strobel G. Li J. Y. Sugawara F. Koshino H. Harper J. Hess W. M. (1999). Oocydin A, a chlorinated macrocyclic lactone with potent anti-oomycete activity from *Serratia marcescens*. *Microbiology* 145 3557–3564. 10.1099/00221287-145-12-3557 10627053

[B107] Swofford D. L. (1993). *PAUP: Phylogenetic analysis using parsimony, version 3.1.1.* Champaign: Illinois Natural History Survey.

[B108] Takahashi S. Tomita J. Nishioka K. Hisada T. Nishijima M. (2014). Development of a prokaryotic universal primer for simultaneous analysis of Bacteria and Archaea using next-generation sequencing. *PLoS One* 9:e105592. 10.1371/journal.pone.0105592 25144201 PMC4140814

[B109] Tank M. Bryant D. A. (2015). *Chloracidobacterium thermophilum* gen. nov., sp. nov.: an anoxygenic microaerophilic chlorophotoheterotrophic acidobacterium. *Int. J. Syst. Evol. Microbiol.* 65 1426–1430. 10.1099/ijs.0.000113 25667398

[B110] Tsaplina O. Demidyuk I. Artamonova T. Khodorkovsky M. Khaitlina S. (2020). Cleavage of the outer membrane protein OmpX by protealysin regulates *Serratia proteamaculans* invasion. *FEBS Lett.* 594 3095–3107. 10.1002/1873-3468.13897 32748449

[B111] Valentine R. C. Valentine D. L. (2004). Omega-3 fatty acids in cellular membranes: a unified concept. *Prog. Lipid Res.* 43 383–402. 10.1016/j.plipres.2004.05.004 15458813

[B112] Varani A. M. Siguier P. Gourbeyre E. Charneau V. Chandler M. (2011). ISsaga is an ensemble of web-based methods for high throughput identification and semi-automatic annotation of insertion sequences in prokaryotic genomes. *Genome Biol.* 12:R30. 10.1186/gb-2011-12-3-r30 21443786 PMC3129680

[B113] Vieira S. Luckner M. Wanner G. Overmann J. (2017). *Luteitalea pratensis* gen. nov., sp. nov. a new member of subdivision 6 *Acidobacteria* isolated from temperate grassland soil. *Int. J. Syst. Evol. Microbiol.* 67 1408–1414. 10.1099/ijsem.0.001827 28141504

[B114] Wang G. Dang G. Xu S. Liu J. Su H. Liang J. (2020). Aliikangiella coralliicola sp. nov., a bacterium isolated from coral *Porites lutea*, and proposal of *Pleioneaceae* fam. nov. to accommodate *Pleionea* and *Aliikangiella*. *Int. J. Syst. Evol. Microbiol.* 70 5880–5887. 10.1099/ijsem.0.004489 33034551

[B115] Wang L. Feng Z. Wang X. Wang X. Zhang X. (2010). DEGseq: an R package for identifying differentially expressed genes from RNA-seq data. *Bioinformatics* 26 136–138. 10.1093/bioinformatics/btp612 19855105

[B116] Wang Y. Tang H. Debarry J. D. Tan X. Li J. Wang X. (2012). MCScanX: a toolkit for detection and evolutionary analysis of gene synteny and collinearity. *Nucleic Acids Res.* 40:e49. 10.1093/nar/gkr1293 22217600 PMC3326336

[B117] Ward N. L. Challacombe J. F. Janssen P. H. Henrissat B. Coutinho P. M. Wu M. (2009). Three genomes from the phylum *Acidobacteria* provide insight into the lifestyles of these microorganisms in soils. *Appl. Environ. Microbiol.* 75 2046–2056. 10.1128/AEM.02294-08 19201974 PMC2663196

[B118] Wu S. Ou H. Liu T. Wang D. Zhao J. (2018). Structure and dynamics of microbiomes associated with the marine sponge *Tedania* sp. during its life cycle. *FEMS Microbiol. Ecol.* 94:5. 10.1093/femsec/fiy055 29617990

[B119] Wu S. Zhu Z. Fu L. Niu B. Li W. (2011). WebMGA: a customizable web server for fast metagenomic sequence analysis. *BMC Genomics* 12:444. 10.1186/1471-2164-12-444 21899761 PMC3180703

[B120] Yarza P. Yilmaz P. Pruesse E. Glöckner F. O. Ludwig W. Schleifer K. H. (2014). Uniting the classification of cultured and uncultured bacteria and archaea using 16S rRNA gene sequences. *Nat. Rev. Microbiol.* 12 635–645. 10.1038/nrmicro3330 25118885

[B121] Yoon S. H. Ha S. M. Lim J. M. Kwon S. J. Chun J. (2017). A large-scale evaluation of algorithms to calculate average nucleotide identity. *Antonie van Leeuwenhoek* 110 1281–1286. 10.1007/s10482-017-0844-4 28204908

[B122] Zhang H. Yohe T. Huang L. Entwistle S. Wu P. Yang Z. (2018). dbCAN2: a meta server for automated carbohydrate-active enzyme annotation. *Nucleic Acids Res.* 46 W95–W101. 10.1093/nar/gky418 29771380 PMC6031026

[B123] Zhang Y. Wang X. Zhen Y. Mi T. He H. Yu Z. (2017). Microbial Diversity and Community Structure of Sulfate-Reducing and Sulfur-Oxidizing Bacteria in Sediment Cores from the East China Sea. *Front. Microbiol.* 8:2133. 10.3389/fmicb.2017.02133 29163420 PMC5682103

[B124] Zimmermann J. Portillo M. C. Serrano L. Ludwig W. Gonzalez J. M. (2012). *Acidobacteria* in freshwater ponds at Doñana National Park, Spain. *Microb. Ecol.* 63 844–855. 10.1007/s00248-011-9988-3 22167078

