# Supplementary material for: Comparative Genomics Reveal the Animal-Associated Features of the Acanthopleuribacteraceae Bacteria, and Description of Sulfidibacter corallicola gen. nov., sp., nov
Source: Front Microbiol. 2022 Jan 31;13:778535. doi: 10.3389/fmicb.2022.778535 (PMC8841776; doi:10.3389/fmicb.2022.778535)
Supplement: Supplementary file 1 [file Data_Sheet_1.PDF]

Supplementary materials

Supplementary Table S5. Cellular fatty acids of strain M133[T] and *Acanthopleuribacter pedis* KCTC 12899[T].

Strains: 1, strain M133[T]; 2, *A. pedis* KCTC 12899[T]. -, less than 1% of the total content; ND, not detected.

Fatty acids	1	2
iso$C_{11:0}$	3.0	4.9
iso$C_{13:0}$	1.5	4.7
$C_{14:0}$	4.7	2.0
$C_{14:1}\omega7c$	2.1	3.6
$C_{15:0}$	8.1	1.8
iso$C_{15:0}$	**35.6**	**17.2**
$C_{16:0}$	**29.8**	**29.6**
$C_{16:0}$ N alcohol	ND	5.8
iso$C_{17:0}$	**3.0**	**12.4**
anteiso$C_{17:1}\omega7c$	-	1.4
$C_{20:0}$cyclo $\omega6c$	-	2.0
$C_{20:5}\omega3c$	**6.2**	**12.2**

Supplementary Table S6. Respiration enzymes of acidobacteria.

A-type, a-type cytochrome c oxidase; cbb3, cbb3 type cytochrome c oxidase; bd, cytochrome d ubiquinol oxidase; a, assimilatory reductase; d, dissimilatory reductase; OmcA/MtrC (F), outer membrane c -type cytochromes; hyd, sulfhydrogenase; -, not detected.

Strains	Electron acceptor						
	oxygen	nitrate	nitrite	Fe(III)/Mn(V)	sulfate	sulfite	sulfur
M133	A-type, cbb3, bd	d	d	-	a	a	hyd
Ape	A-type, cbb3, bd	d	d	-	a	a	-
Gfe	A-type, bd	d	d	MtrF	d	a	-
Hfo	A-type, bd	-	d	-	a	a, d	-
Cth	Cbb3	-	-	-	-	-	-
Lpr	A-type, cbb3	a	d	-	d	-	-
Pme	A-type, bd	a	a	-	a	-	-
Taq	A-type	d	-	OmcA/MtrC	-	-	-
Aca	A-type, cbb3,bd	-	a	-	a	a	-
Apo	A-type	-	-	-	a	a	-
Aro	A-type,bd	-	-	-	a	a	-
Bel	A-type, bd	-	-	-	a	a	-
Emo	A-type, bd	-	-	-	a	a	-
Gpe	A-type	-	-	-	a	a	-
Osa	A-type, cbb3, bd	-	a	-	a	-	-
Sbo	A-type,bd,cbb3	-	-	-	-	a	-
Tga	A-type	-	-	-	-	a	-
Tro	A-type, cbb3	a	a	-	a	a	-
Bag	A-type	-	-	-	-	a	-
Pfe	A-type,bd,cbb3	a	a	OmcA/MtrC	-	a	-
CSu	A-type, bd	a	a	OmcA/MtrC	a	a	-

Taxonomy abbreviation

Ape	*Acanthopleuribacter pedis* KCTC 12899^T	Bel	*Bryocella elongata* DSM 22489^T
Gfe	*Geothrix fermentans* DSM 14018^T	Emo	*Edaphobacter modestus* DSM 18101^T
Hfo	*Holophaga foetida* DSM 6591^T	Gpe	*Granulicella pectinivorans* DSM 21001^T
Cth	*Chloracidobacterium thermophilum* B^T	Osa	*Occallatibacter savannae* AB23^T
Lpr	*Luteitalea pratensis* DSM 100886^T	Sbo	*Silvibacterium bohemicum* S15^T
Pme	*Pyrinomonas methylaliphatogenes* K22^T	Tga	*Terracidiphilus gabretensis* S55^T
Taq	*Thermoanaerobaculum aquaticum* MP-01^T	Tro	*Terriglobus roseus* DSM 18391^T
Aca	*Acidobacterium capsulatum* ATCC 51196^T	Bag	*Bryobacter aggregatus* MPL3^T
Apo	'Acidisarcina polymorpha' SBC82	Pfe	*Paludibaculum fermentans* P105^T
Aro	*Acidipila rosea* DSM 103428^T	CSu	*Candidatus* Solibacter usitatus Ellin6076

Supplementary Table S7. Sulfhydrogenase from bacteria and archaeon.

No.	Strains	(G+C) mol%		Phylum/Class
		Genome	*hydABCD*	
1	M133(CP071793)	60.2	63.92	*Acidobacteria*
2	*Acidobacteria* bacterium SZAS-15 (JAFDVL010000014)	51.5	54.41	
3	*Blastocatellia* bacterium MAG-251 (JAEUQM010000315)	34.5	36.77	*Cyanobacteria*
4	*Leptolyngbya boryana* PCC 6306 (KB731325)	47	47.39	
5	*Mastigocoleus testarum* BC008 (LMTZ01000055)	42.75	42.21	
6	*Caldilinea aerophila* DSM 14535(NC_017079)	58.8	62.39	*Chloroflexi*
7	*Meiothermus cerbereus* DSM 11376 (JHVI01000016)	61.4	64.14	*Deinococcus-Thermus*
8	*Nitrospina gracilis* 3/211 (HG422173)	56.1	60.74	*Nitrospinae*
9	*Methylosinus sporium* SM89A (VJMF01000146)	64.5	65	*Alphaproteobacteria*
10	*Methylocystis rosea* SV97 (KB889963)	62.5	63.76	
11	*Beijerinckia indica* subsp. *indica* ATCC 9039 (NC_010581)	57.1	59.74	
12	*Tistlia consotensis* USBA 355(FWZX01000010)	70.95	74.08	
13	*Novosphingobium indicum* CGMCC 1.6784 (BMLK01000005)	63.1	62.79	
14	*Pseudoruegeria marinistellae* SF-16 (LNCI01000004)	63	66.89	
15	*Paraburkholderia phytofirmans* OLGA172 (CP014580)	61.58	63.84	*Betaproteobacteria*
16	*Nitrosomonas* sp. Nm143 (QLTE01000005)	43.2	47.21	
17	*Polyangium fumosum* DSM 14668 (SSMQ01000030)	68.8	66.91	*Deltaproteobacteria*
18	*Nitrococcus mobilis* Nb-231 (CH672427)	59.9	63.77	*Gammaproteobacteria*
19	*Azotobacter salinestris* KACC 13899 (CP045302)	65.60	70.46	
20	*Halomonas azerbaijanica* TBZ202 (SBLD01000005)	67.3	72.51	
21	*Sinimarinibacterium* sp. CAU 1509 (SUKF01000006)	63.2	65.71	
22	*Marinobacter lipolyticus* SM19 (KE007306)	56.8	60.49	
23	*Rhodothermus marinus* AA2-13 (AP019796)	64.1	67.16	*Bacteroidetes*
24	*Mucilaginibacter gotjawali* SA3-7 (AP017313)	43.2	46.85	
25	*Schlesneria paludicola* DSM 18645 (JH636435)	55.6	54.76	*Planctomycetes*
26	*Methylacidimicrobium tartarophylax* 4A (CABFVA020000066)	61.2	69.04	*Verrucomicrobia*
27	*Actinobacteria* bacterium 5_8_m_bin21 (JAENWQ010000023)	64.7	63.26	*Actinobacteria*
28	*Nocardiopsaceae* bacterium Bacteria.bin_25 (JAFMRD010000008)	71.7	72.25	
29	*Pyrococcus furiosus* DSM 3638 (CP023154)	40.8	43.84	*Euryarchaeota*
30	*Thermococcus barophilus* CH5 (CP013050)	41.8	44.17	
31	Archaeon J079 (RFIP01000354)	33.1	32.84	*Thaumarchaeota*

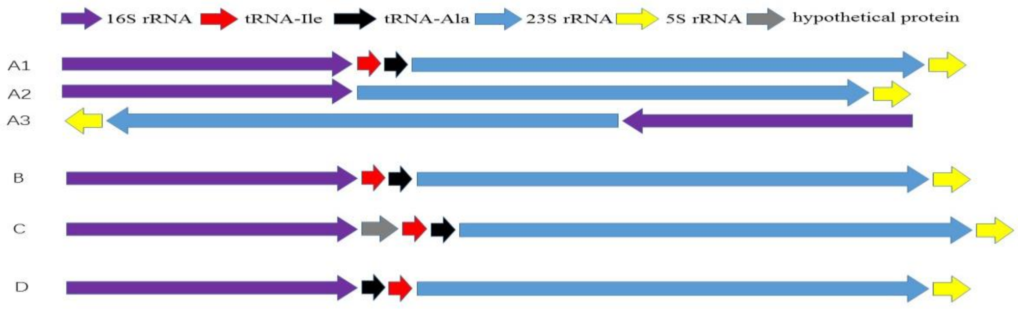

Supplementary Figure S1. Patterns of rRNA operons in acidobacteria. A1-3, rRNA operons of strain M133^T, A1 is ortholog, A3 is reverse with tRNA deletion; B, rRNA operons of other acidobacteria; C, rRNA operons of 'Acidisarcina polymorpha' SBC82, with hypothetical protein insertion before tRNA-Ile; D, rRNA operons for *Bryobacteraceae* bacteria with reverse insertion of tRNAs.

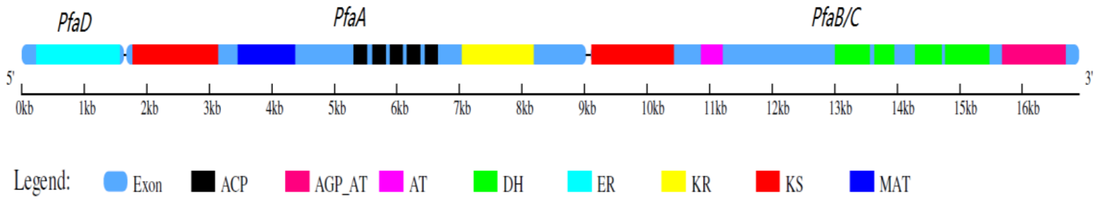

Supplementary Figure S2. Domain architecture of *Pfa* for eicosapentaenoic acid de novo biosynthesis. ACP, Acyl carrier protein; AT, acyltransferase; DH, Dehydrase/isomerase; ER, Enoyl reductase; KR, Ketoacyl reductase; KS, Beta-ketoacyl synthase; MAT, Malonyl-CoA : ACP transacylase. AGP_AT, 1-Acyl-sn-glycerol-3-phosphate acyltransferase.

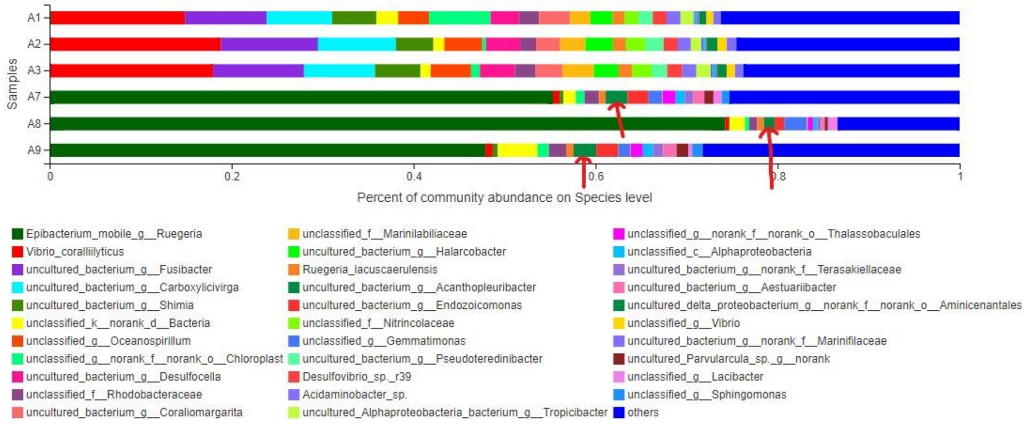

Supplementary Figure S3. Coral *Porites lutea* bacterial composition change after three months of axenic incubation. A1-3, the initial coral sample; A7-9, coral sample after three months of axenic incubation (seawater was sterilized, no feeding). Red arrows show the obvious increasing of *Acanthopleuribacter* sp. OTU abundance.

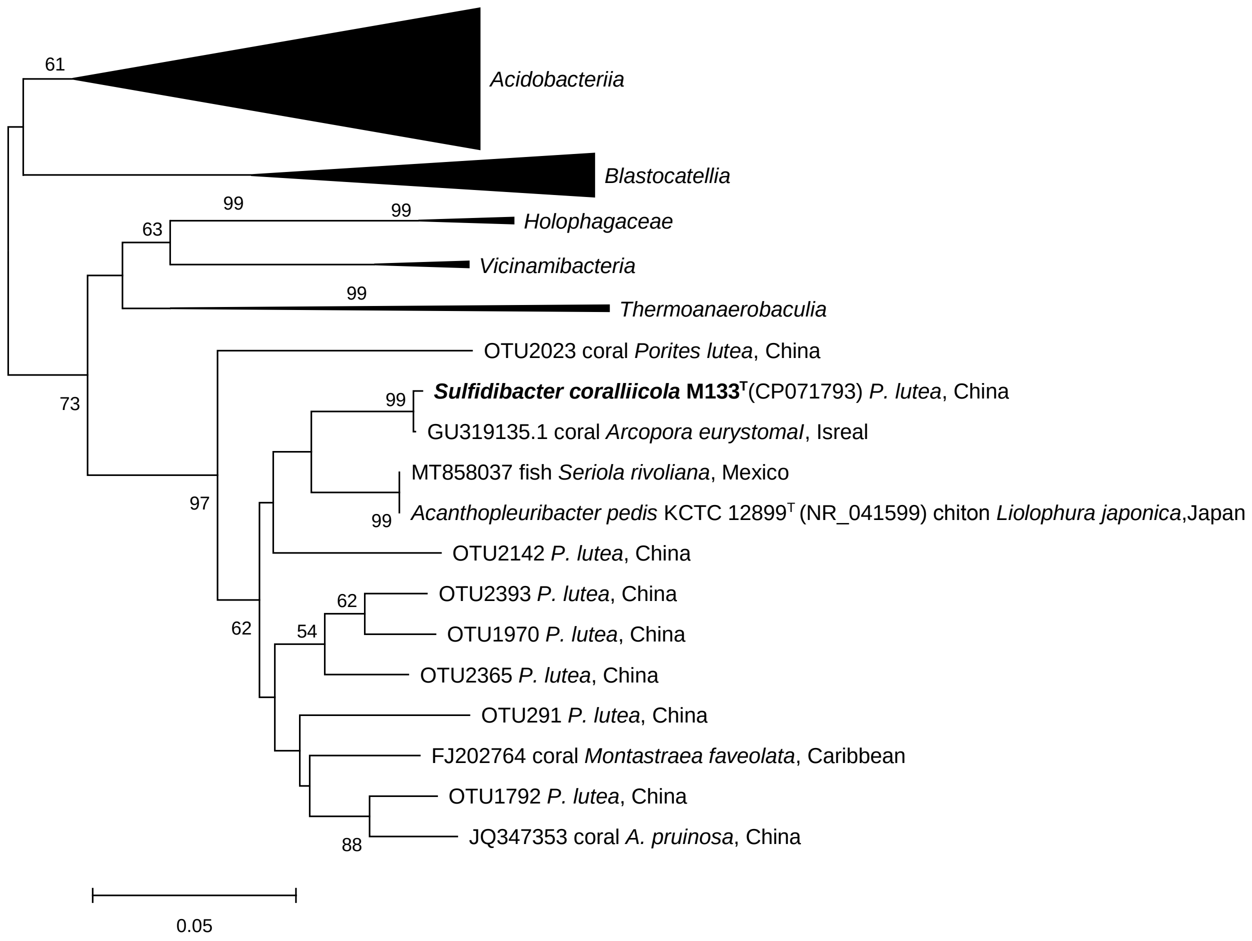

Supplementary Figure S4. Neighbor-joining phylogenetic tree based on the 16S rRNA gene sequences indicated almost all strain M133^T allied OTUs or species are from marine animals, especially coral. Markers after OTUs or GenBank accession No. are hosts and sample locations. Numbers at nodes indicate percentages of 1000 bootstrap resamplings, only values above 50% are shown. Bar, 0.05 substitutions per nucleotide position.

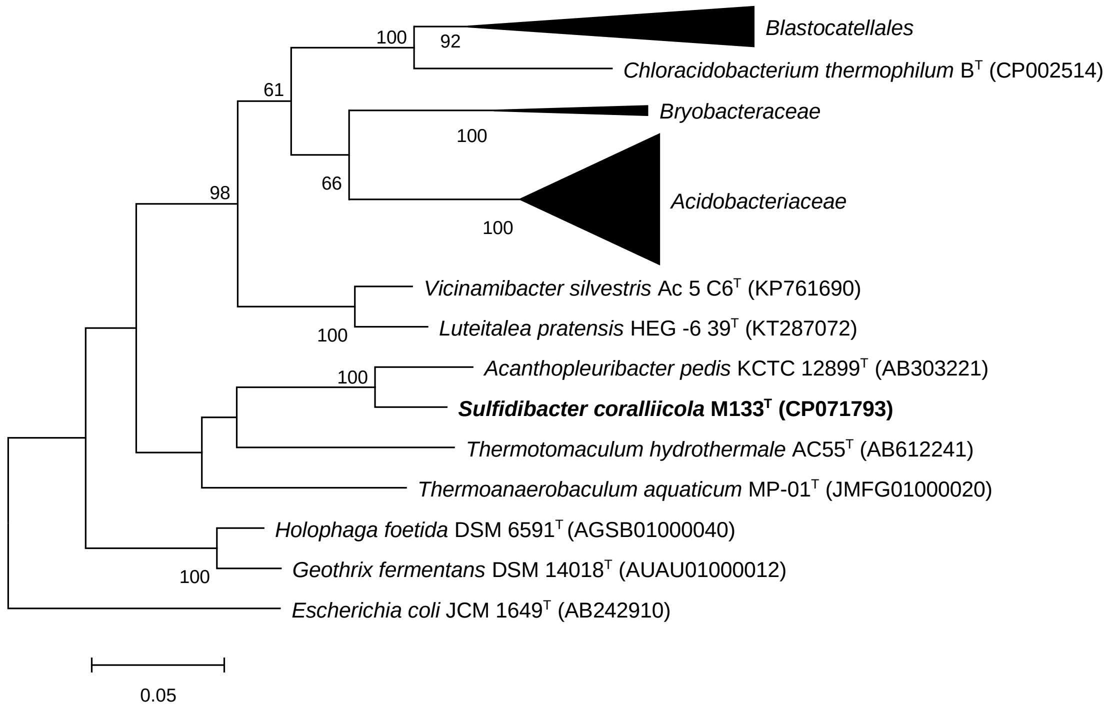

Supplementary Figure S5. Maximum likelihood phylogenetic tree based on the 16S rRNA gene sequences of the acidobacteria. Sequences from the *Escherichia coli* was used as out group. Numbers at nodes indicate percentages of 1000 bootstrap resamplings, only values above 50% are shown. Bar, 0.05 substitutions per nucleotide position.

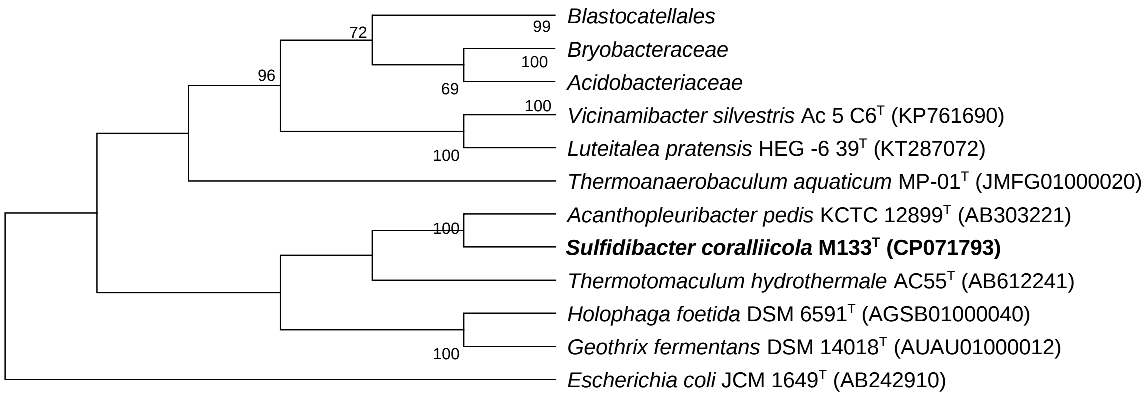

Supplementary Figure S6. Maximum-parsimony phylogenetic tree based on the 16S rRNA gene sequences of acidobacteria. Numbers at nodes indicate percentages of 1000 bootstrap resamplings, only values above 50% are shown.

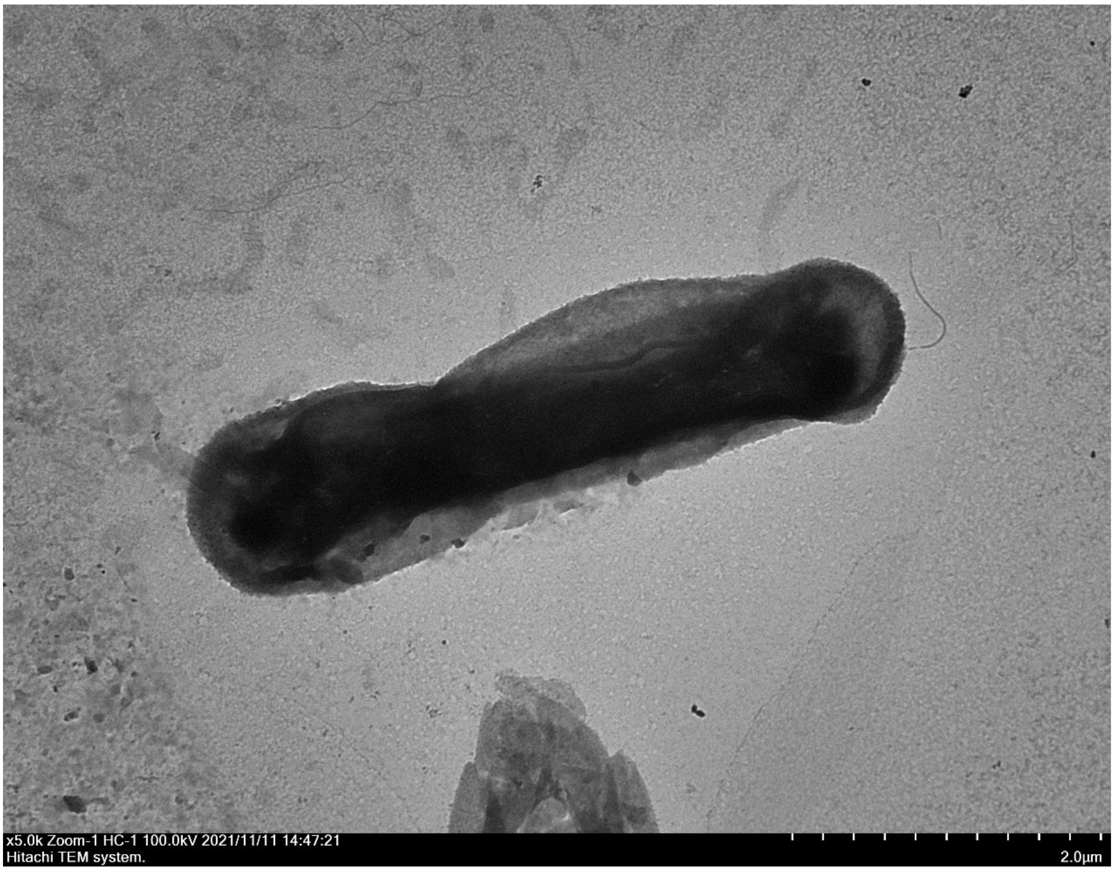

Supplementary Figure S7. Transmission electronic microscopy of strain M133^T displayed the single polar flagellum. Bar, 2 μm.

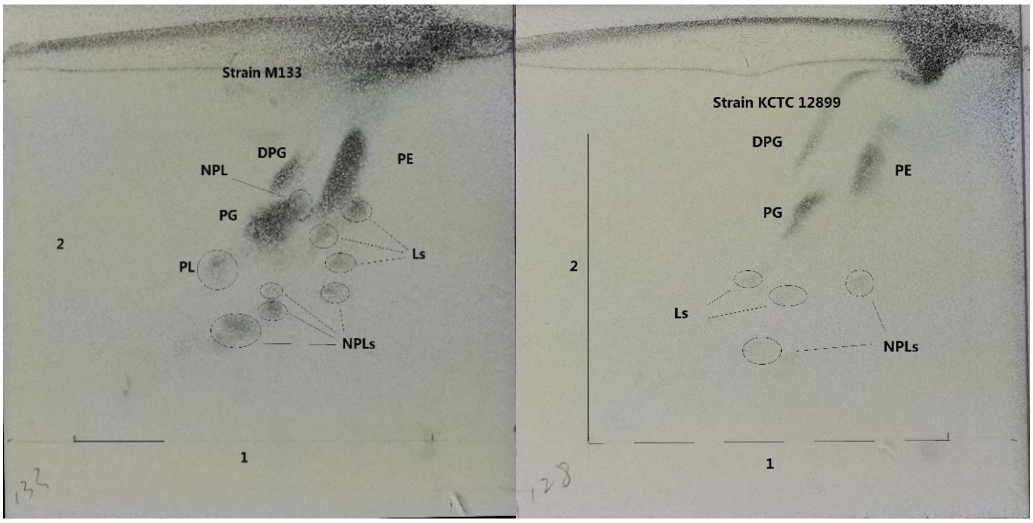

Supplementary Figure S8. The two-dimensional thin-layer chromatography of polar lipids of strains M133^T and *A. pedis* KCTC 12899^T. Polar lipids were sprayed using phosphomolybdic acid, ninhydrin, and molybdenum blue reagent. PE, phosphatidylethanolamine; PG, phosphatidylglycerol; DPG, diphosphatidylglycerol; PL, unidentified phospholipid, NPLs, unidentified ninhydrin positive lipids; L, unidentified lipids.
